# Novel hypoxia- and lactate metabolism-related molecular subtyping and prognostic signature for colorectal cancer

**DOI:** 10.1186/s12967-024-05391-5

**Published:** 2024-06-20

**Authors:** An Huang, Zhuang Sun, Haopeng Hong, Yong Yang, Jiajia Chen, Zhaoya Gao, Jin Gu

**Affiliations:** 1 Key Laboratory of Carcinogenesis and Translational Research (Ministry of Education/Beijing), Department of Gastrointestinal Surgery III, Peking University Cancer Hospital & Institute, Haidian District, Beijing, 100142 China; 2 Department of Gastrointestinal Surgery, Peking University Shougang Hospital, Beijing, 100144 China; 3 Department of General Surgery, Peking University First Hospital, Beijing, 100034 China

**Keywords:** Colorectal cancer, Hypoxia, Lactate metabolism, Molecular subtype, Prognosis, Immune microenvironment

## Abstract

**Background:**

Colorectal cancer (CRC) is a serious global health burden because of its high morbidity and mortality rates. Hypoxia and massive lactate production are hallmarks of the CRC microenvironment. However, the effects of hypoxia and lactate metabolism on CRC have not been fully elucidated. This study aimed to develop a novel molecular subtyping based on hypoxia-related genes (HRGs) and lactate metabolism-related genes (LMRGs) and construct a signature to predict the prognosis of patients with CRC and treatment efficacy.

**Methods:**

Bulk and single-cell RNA-sequencing and clinical data of CRC were downloaded from the TCGA and GEO databases. HRGs and LMRGs were obtained from the Molecular Signatures Database. The R software package DESeq2 was used to perform differential expression analysis. Molecular subtyping was performed using unsupervised clustering. A predictive signature was developed using univariate Cox regression, random forest model, LASSO, and multivariate Cox regression analyses. Finally, the sensitivity of tumor cells to chemotherapeutic agents before and after hypoxia was verified using in vitro experiments.

**Results:**

We classified 575 patients with CRC into three molecular subtypes and were able to distinguish their prognoses clearly. The C1 subtype, which exhibits high levels of hypoxia, has a low proportion of CD8 + T cells and a high proportion of macrophages. The expression of immune checkpoint genes is generally elevated in C1 patients with severe immune dysfunction. Subsequently, we constructed a predictive model, the HLM score, which effectively predicts the prognosis of patients with CRC and the efficacy of immunotherapy. The HLM score was validated in GSE39582, GSE106584, GSE17536, and IMvigor210 datasets. Patients with high HLM scores exhibit high infiltration of CD8 + exhausted T cells (Tex), especially terminal Tex, and oxidative phosphorylation (OXPHOS)−Tex in the immune microenvironment. Finally, in vitro experiments confirmed that CRC cell lines were less sensitive to 5-fluorouracil, oxaliplatin, and irinotecan under hypoxic conditions.

**Conclusion:**

We constructed novel hypoxia- and lactate metabolism-related molecular subtypes and revealed their immunological and genetic characteristics. We also developed an HLM scoring system that could be used to predict the prognosis and efficacy of immunotherapy in patients with CRC.

**Supplementary Information:**

The online version contains supplementary material available at 10.1186/s12967-024-05391-5.

## Background

Colorectal cancer (CRC) is currently the third most common cancer and the second leading cause of cancer-related deaths worldwide [1]. Growing evidence suggests that the tumor microenvironment (TME) plays an important role in the development, progression, and drug resistance of CRC.

Hypoxia is a prominent feature of the microenvironments of CRC and other solid tumors. The generation of hypoxic conditions may be related to the massive consumption of oxygen due to uncontrolled tumor proliferation and impaired oxygen supply due to irregular and disorganized tumor-associated neovascularization. Hypoxic conditions increase the levels of hypoxia-inducible factors (HIFs) in cells. As transcription factors, HIFs further increase the transcription of downstream target genes, thus playing a regulatory role in various biological processes, such as cell metabolism, proliferation, metastasis, epithelial-mesenchymal transition (EMT), and angiogenesis [2–10]. Additionally, hypoxia and HIF accumulation have been found to be associated with resistance to chemotherapy as well as worse prognosis in patients with CRC [11–14]. Recent studies have also found that hypoxia can be considered a biomarker for predicting the outcome of immunotherapy and that the efficacy of immunotherapy can be improved by ameliorating hypoxia and targeting HIF-1 [15–19]. Several studies have demonstrated that hypoxia regulates the function and differentiation of immunosuppressive cells, such as myeloid-derived suppressor cells, tumor-associated macrophages (TAMs), and regulatory T cells (Tregs), thereby promoting immunosuppression and tumor immune escape [20–25].

The hypoxia-mediated shift of tumor metabolism towards glycolysis, as well as the native characteristics of aerobic glycolysis (the so-called Warburg effect), leads to massive glucose consumption by tumor cells, consequently increasing lactate production and decreasing the pH of the TME [26]. Lactate can directly modulate endothelial cell phenotype and drive tumor angiogenesis through multiple pathways [27–29]. Furthermore, considerable lactate accumulation stimulates macrophage polarization to the M2 phenotype in tumors [30]. Lactate has been found to reduce interferon-γ (IFN-γ) production by CD8 + T cells and NK cells, inhibiting their cytotoxic function [31]. Lactate also decreases the motility of CD4 + and CD8 + T cells, which might reduce their infiltration and movement into the TME [32]. Additionally, lactate in the highly glycolytic TME may increase the expression of programmed cell death-1 (PD-1) in Tregs; PD-1 blockade therapy could activate PD-1-expressing Tregs, leading to immunotherapy failure [33]. Lactate has also recently been found to provide metabolic support to tumor-infiltrating Tregs [34]. Moreover, patients with metastatic CRC have been reported to have higher serum lactate concentrations than those with non-metastatic CRC [35].

However, considering the heterogeneity of CRC and the complex interaction between hypoxia and lactate metabolism, the effects of hypoxia and lactate metabolism on CRC have not been fully elucidated. Therefore, it is necessary to conduct a landscape assessment of the fundamental combination of hypoxia and lactate metabolism on CRC prognosis, TME, and immunotherapy. In this study, we performed a new subtype classification of CRC by combining hypoxia-related genes (HRGs) and lactate metabolism-related genes (LMRGs). We then identified the characteristics of the corresponding subtypes from multiple perspectives. Overall, this study aimed to determine a precise treatment strategy for CRC using this new subtype classification method, thereby improving the treatment efficacy and survival of patients with CRC.

## Methods

### Data acquisition

We obtained RNA sequencing data of CRC, as well as clinical information from the TCGA database (https://portal.gdc.cancer.gov/). After removing patients with less than 1 month of follow-up, we included count and transcripts per million (TPM) data from 575 patients with CRC and 51 normal tissues. The GSE39582, GSE106584, and GSE17536 expression matrices and clinical information were downloaded from the GEO database (https://www.ncbi.nlm.nih.gov/). The IMvigor210 data were obtained from IMvigor210CoreBiologies (http://research-pub.gene.com/IMvigor210CoreBiologies). HRGs and LMRGs were obtained from the Molecular Signatures Database (http://www.gsea-msigdb.org/gsea/downloads.jsp). Specifically, 326 HRGs were identified from Harris hypoxia, hallmark hypoxia, GOBP regulation of cellular responses to hypoxia, and reactome cellular responses to hypoxia. Overall, 288 LMRGs were obtained from the GOBP glucose catabolic process to lactate via pyruvate, GOBP lactate metabolic process, GOBP lactate transmembrane transport, GOMF L-lactate dehydrogenase activity, GOMF lactate dehydrogenase activity, GOMF lactate transmembrane transporter activity, HP abnormal brain lactate level by mrs, HP abnormal lactate dehydrogenase level, HP-elevated lactate pyruvate ratio, HP-increased circulating lactate dehydrogenase concentration, HP-increased CSF lactate level, and HP-increased serum lactate level.

### Differential expression analysis

The R software package DESeq2 (version 1.32.0) was used for differential expression analysis. Genes with a false discovery rate (FDR) < 0.05 and |fold change|≥ 2 were screened as differentially expressed genes (DEGs). We analyzed the correlations among the 35 DEGs using Spearman correlation analysis. GeneMANIA was used to assess gene interactions and predict gene functions [36]. Additionally, we evaluated the prognostic significance of each gene using the Cox method with the survival package (version 3.5-5), which integrates survival time, survival status, and gene expression data.

### Molecular subtyping

Cluster analysis was performed using ConsensusClusterPlus [37] with agglomerative PAM clustering with Euclidean distance; 80% of the samples were resampled for 10 repetitions. The optimal number of clusters was determined using an empirical cumulative distribution function plot and the average consistency within the group.

The prognostic differences between the groups were analyzed using the Survfit function of the R package survival (version 3.5-5). The log-rank test was used to assess the statistical significance, and Kaplan–Meier curves were plotted.

We classified the CRC samples in the TCGA database into four consensus molecular subtypes (CMS), following the report of Guinney et al. [38]. Gene Set Enrichment Analysis (GSEA) software (version 3.0) was utilized to perform GSEA. The h.all.v2023.1.Hs.symbols.gmt subset of the MSigDB was downloaded to assess the related pathways and molecular mechanisms.

### Immune landscape analysis

We calculated 22 immune cell infiltration profiles per sample based on the CIBERSORT method in the R package IOBR [39]. Next, we analyzed and visualized the anticancer immune status of each sample and the proportion of tumor-infiltrating immune cells in the seven-step cancer immune cycle based on RNA-seq data in the Tracking Tumor Immunophenotype (TIP, http://biocc.hrbmu.edu.cn/TIP/) [40]. The immunophenoscore (IPS) and Tumor Immune Dysfunction and Exclusion (TIDE) score were used to assess the potential clinical efficacy of immunotherapies in the different subtypes and indicate the potential for tumor immune evasion. The IPS and TIDE scores for the samples were derived from The Cancer Immunome Atlas (TCIA, https://tcia.at/home/) and an online tool called TIDE (http://tide.dfci.harvard.edu/), respectively [41]. Finally, we compared the expression levels of well-known immune checkpoint genes across the three subtypes.

### Prediction of patients’ clinical drug response

The R package oncoPredict (version 0.2) [42] was used to predict each patient’s response to multiple clinical medications based on cell line drug response and gene expression data from the Broad Institute’s Cancer Therapeutics Response Portal (CTRP) and Sanger Genomics of Drug Sensitivity in Cancer (GDSC).

### Characterization of somatic mutations in patients

To characterize the somatic mutations in patients with different subtypes, mutation annotation format (MAF) files of patients were downloaded from the TCGA database. These files were analyzed and visualized using the R package maftools (version 2.10.05) [43].

### Construction and validation of the prognostic model

Differential expression analysis was performed as previously described. Kyoto Encyclopedia of Genes and Genomes (KEGG) and Gene Ontology (GO) enrichment analyses were performed using the R package clusterProfiler (version 3.14.3) to obtain the results of the gene set enrichment. The KEGG Rest API (https://www.kegg.jp/kegg/rest/keggapi.html) was also used. Genes in the R package org.Hs.eg.db (version 3.1.0) were used for GO annotation.

The R package randomForest (version 4.7-1.1) was used to develop optimal models. The importance of each explanatory variable was assessed by evaluating the mean decrease in accuracy and the Gini coefficient. The top 20 genes in terms of importance were selected for the least absolute shrinkage and selection operator (LASSO) regressions. The R package glmnet (version 4.1-8) was used to perform the LASSO analysis. A stepwise multivariate Cox proportional hazards model was used to optimize the model. Finally, we constructed an HLM scoring system based on six genes for prognostic prediction. HLM score = 0.43*(AGXT expression) + 0.03*(TRIB2 expression) + 0.17*(ELFN2 expression) + 0.16*(PCDHB10 expression) + 0.02*(CALCA expression) + 0.01*(CD79A expression). To validate the efficacy of the model, receiver operating characteristic (ROC) analysis was conducted at one, three, and five years using the R package Proc (version 1.17.0.1). The area under the curve (AUC) and confidence intervals were evaluated using the ci function of Proc.

To validate the stability of our model, we acquired GSE39582, GSE106584, GSE17536, and IMvigor210 expression matrices and clinical information. These data were divided into the high- and low-risk groups based on the HLM score using optimal truncation, and survival analysis was performed.

### Single-sample gene set enrichment analysis (ssGSEA)

We obtained and defined the CD8 + exhausted T cell (Tex), GZMK + Tex, terminal Tex, oxidative phosphorylation (OXPHOS)−Tex, and TCF7 + Tex gene sets from previous studies (Supplementary Table 1) [44, 45], set the minimum gene set to 5 and the maximum gene set to 5000, and calculated the enrichment scores for each sample in each gene set using the R package GSVA (version 1.40.1).

### Single-cell RNA sequencing (scRNA-seq) data processing and analysis

The scRNA-seq data used in this study were downloaded from the GEO database under the accession code GSE132465. This dataset contains single-cell 3'-RNA sequencing data from 23 patients with primary CRC [46]. The Seurat R package (version 5.0) was used to analyze scRNA-seq data according to standard analysis procedures [47]. Cells were selected for analysis based on the following criteria: cells with unique molecular identifier (UMI) counts greater than 1000, cells with more than 200 but less than 6000 unique genes, and cells with less than 20% mitochondrial gene expression in their UMI counts. The ElbowPlot function was used to determine the dimensionality of each dataset. The t-distributed stochastic neighbor embedding (t-SNE) projection was used to visualize the cell clusters. Major cell types and subtypes were annotated by comparing the typical marker genes and differentially expressed genes in each cluster.

### Pseudo-bulk analysis of scRNA-seq data

Pseudo-bulk analysis of scRNA-seq data was performed to characterize the gene expression profile of each sample. Specifically, scRNA-seq data were converted to bulk-like data by aggregating the gene counts of individual cells belonging to each sample.

### Construction of a predictive nomogram

To visualize the prognosis prediction in patients with CRC, we constructed a nomogram based on survival time, survival status, age, sex, T stage, N stage, M stage, pathological type, and HLM score using the R package rms (version 6.7-1). Harrell’s concordance index (C-index), calibration curves, and decision curve analysis (DCA) were used to assess the nomogram performance.

### Protein interactions

Protein interactions mediate a range of physiological functions and pathological developments in organisms. We obtained the interaction networks of proteins that interacted with the characterized genes from the BioPlex Interactome database (https://bioplex.hms.harvard.edu/) [48]. The purple circles represent the quered protein, the green circles represent the bait protein, the gray diamonds represent the prey protein, and the arrows represent the directed edge (bait-to-prey).

### Cell viability assay

The HCT116 and LS174T cell lines were purchased from the American Type Culture Collection (ATCC) website. The cells were cultured in 1640 medium (Invitrogen, C11875500BT) containing 10% fetal bovine serum (Vivacell, C04001-500) and 1% penicillin and streptomycin (Gibco, 15140122) at 37 °C in a 5% CO_2_ incubator. A hypoxic environment was created using a CO_2_ three-gas incubator (Thermo Fisher Scientific, 51901137); the O_2_ concentration was adjusted to 1%.

We seeded HCT116 and LS174T cells in 96-well plates (Costar 3599) at densities of 8,000 and 15,000 cells/well, respectively, and placed them in normoxic or hypoxic environments. The following day, different concentrations of 1640 complete medium, 5-fluorouracil (5-FU, Solarbio, F8300), oxaliplatin (Solarbio, O8390), or irinotecan (Solarbio, II0140), in combination with or without BAY87-2243 (a potent and selective HIF-1 inhibitor; Beyotime, SC1193), were added to the 96-well plates. The cells were then incubated under normoxic or hypoxic environments for another 48 h.

For Trypan blue staining, the spent medium in the wells was discarded, 100 µl of 0.4% Trypan blue (LABLEAD, T6146) solution was added to each well, and then the Trypan blue stain was washed away with PBS (Invitrogen, C20012500BT) after 5 min. Finally, the cells were observed and photographed under the microscope.

For Cell Counting Kit-8 (CCK8) experiments, the spent medium in each well of the 96-well plate was discarded, 100 µl of 1640 medium containing 10% CCK8 (Beyotime, C0038) was added to each well, and the plates were incubated at 37 °C for 2 h. Finally, the absorbance at 450 nm was measured using an enzyme labeling instrument (Thermo Fisher Scientific, VLBL00D0).

### Statistical analysis

SPSS 26.0 (SPSS, Inc., Chicago, IL, USA) and GraphPad Prism 8 (GraphPad, Inc., CA, USA) software were used for the analyses. Differences between two groups were calculated using the paired two-tailed Student’s t-test or the Mann–Whitney–Wilcoxon test. Comparisons among three groups were performed using ANOVA or the Kruskal–Wallis rank-sum test. The chi-square test was used to compare the clinical characteristics. Statistical significance was set at p < 0.05.

## Results

### Identification of hypoxia-associated DEGs and lactate metabolism-associated DEGs in CRC

**Fig. 1:**
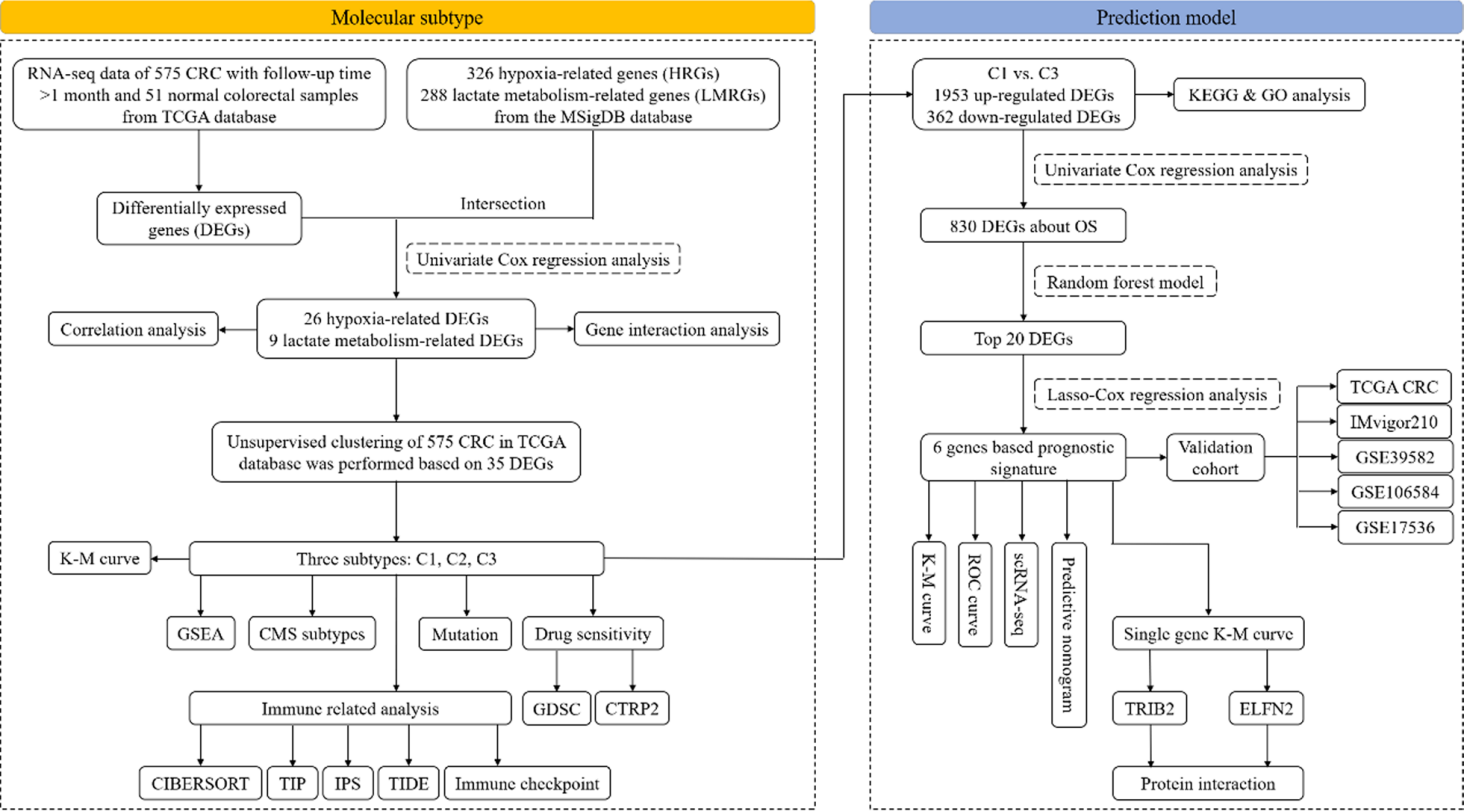
Outline of the analyses performed in this study. CRC: colorectal cancer; TCGA: The Cancer Genome Atlas; K–M: Kaplan–Meier; GSEA: gene set enrichment analysis; CMS: consensus molecular subtype; KEGG: Kyoto Encyclopedia of Genes and Genomes; GO: Gene Ontology; LASSO: least absolute shrinkage and selection operator; ROC: receiver operating characteristic; scRNA-seq, single-cell RNA-seq

**Fig. 2:**
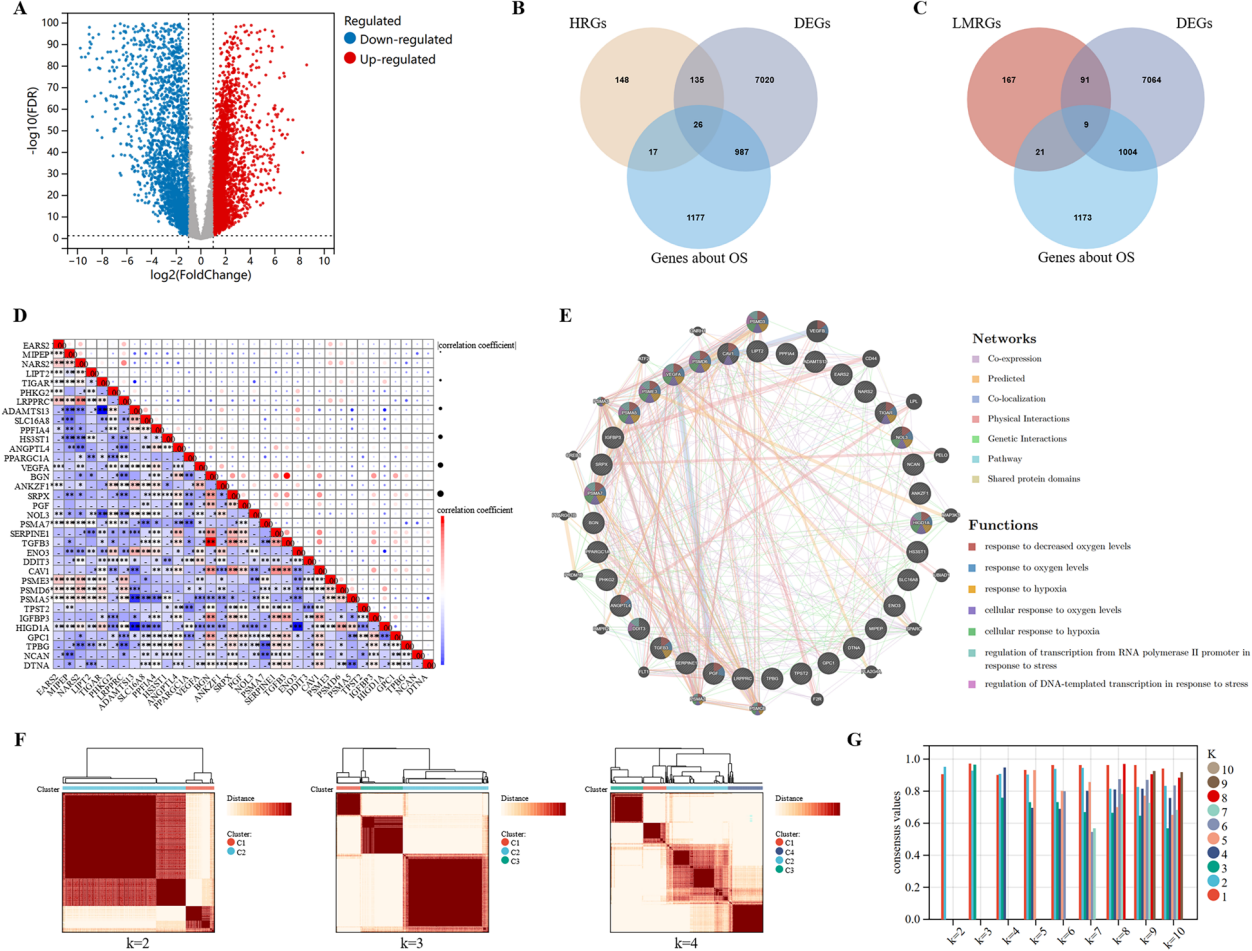
Identification of DEHRGs and DELMRGs in CRC. **A** Volcano plot of the DEGs between CRC and normal tissues. **B** Venn diagram of the HRGs, DEGs, and genes related to OS. **C** Venn diagram of the LMRGs, DEGs, and genes related to OS. **D** Heatmap of the correlations between the 35 DEHRGs and DELMRGs. **E** Interactions and gene function of the 35 DEHRGs and DELMRGs. **F** Heatmap for different numbers of clusters after unsupervised clustering. **G** Within-group clustering consistency at different numbers of clusters. DEHRGs: different expressed hypoxia-related genes; DELMRGs: different expressed lactate metabolism-related genes; CRC: colorectal cancer; OS: overall survival

Figure 1 illustrates the analytical framework used in this study. RNA-seq data were obtained from the TCGA database for 575 CRC samples with more than 1 month of follow-up, and 51 normal colorectal tissues were used for comparison. Differential expression analysis was performed, and 8168 DEGs were identified in CRC when the threshold was set at |fold change|> 2 and FDR < 0.05. Of these genes, 4496 were upregulated and 3672 were downregulated (Fig. 2A). Additionally, 326 HRGs and 288 LMRGs were retrieved from the MSigDB database. We then conducted univariate Cox regression analysis on all protein-coding genes in the RNA-seq data of patients and identified 2207 genes associated with overall survival (OS). After taking the intersection of DEGs, genes associated with OS, HRGs, and LMRGs, we identified 26 hypoxia-related DEGs (HRDEGs) and 9 lactate metabolism-related DEGs (LMRDEGs, Fig. 2B, C). Analysis of these 35 genes revealed a wide range of correlations and intergene interactions (Fig. 2D, E).

### Constructing molecular subtypes based on the HRDEGs and LMRDEGs

**Fig. 3:**
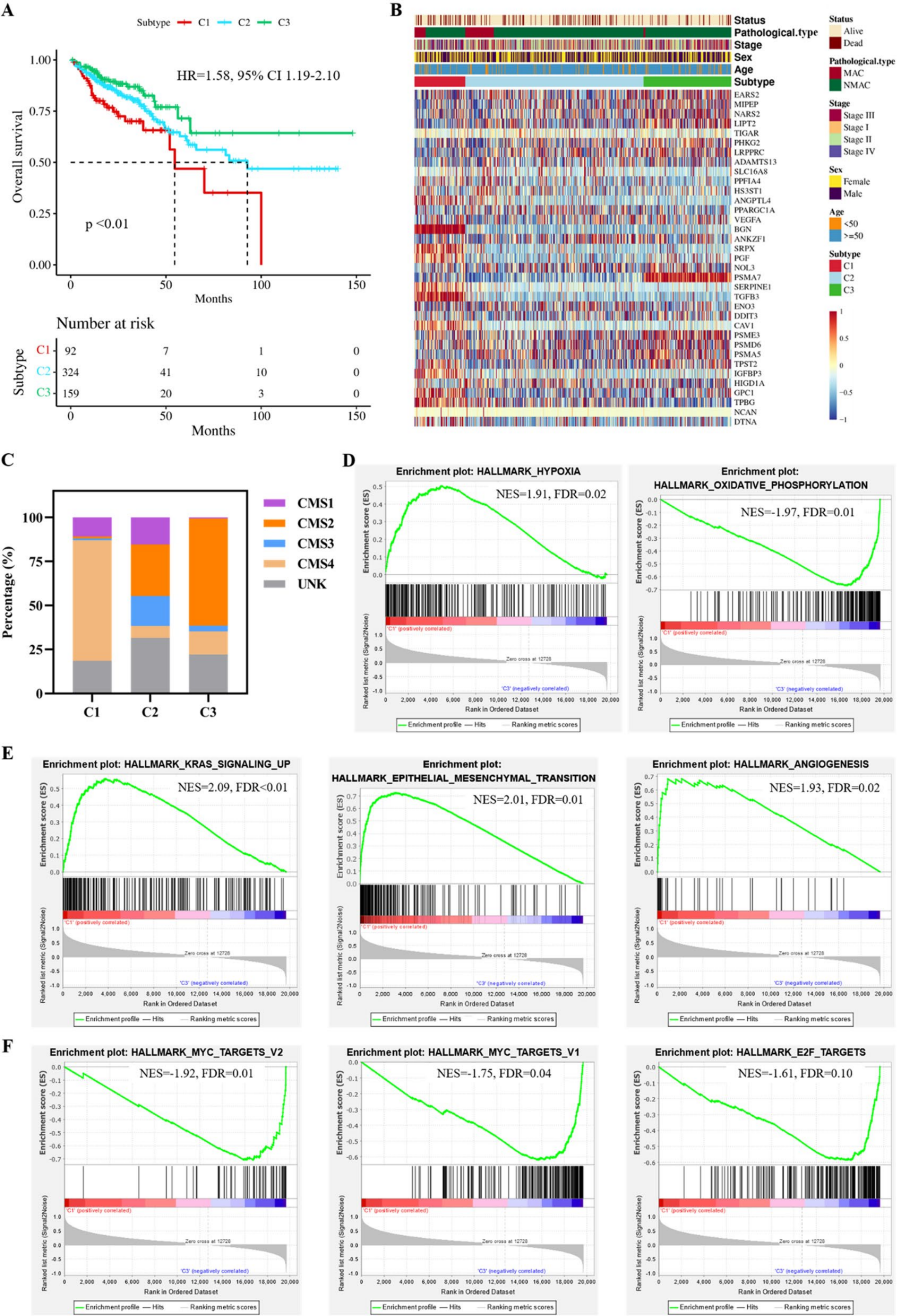
Molecular subtyping based on the DEHRGs and DELMRGs. **A** Kaplan–Meier curve of the different molecular subtypes. **B** Heatmap of the 35 DEHRGs and DELMRGs in different subtypes and their association with clinical characteristics. **C** Distribution of the CMS in the molecular subtypes based on the DEHRGs and DELMRGs. **D** The hypoxia pathway was significantly upregulated and the oxidative phosphorylation pathway was downregulated in the C1 subtype. **E** The top 3 HALLMARK signaling pathways upregulated in the C1 subtype than in the C3 subtype, except hypoxia. **F** The top 3 HALLMARK signaling pathways upregulated in the C3 subtype than in the C1 subtype, except hypoxia. DEHRGs: different expressed hypoxia-related genes; DELMRGs: different expressed lactate metabolism-related genes; CMS: consensus molecular subtype; MAC: mucinous adenocarcinoma; NMAC: non-mucinous adenocarcinoma

Unsupervised clustering was performed on 575 CRC samples using the 35 identified HRDEGs and LMRDEGs. The highest average within-group agreement was observed when classified into three subtypes (Fig. 2F, G). Therefore, we classified the 575 samples into three subtypes: C1 (n = 92), C2 (n = 324), and C3 (n = 159). A comprehensive evaluation revealed that different molecular subtypes exhibited distinct hypoxia and lactate metabolism-related microenvironments (Figure S1A, B). The Kaplan–Meier survival curves indicated that patients with the C1 subtype had the worst OS than patients with subtypes C2 (HR = 1.67, 95% CI 1.07–2.62, p = 0.02) and C3 (HR = 2.48, 95% CI 1.41–4.36, p < 0.01). Additionally, there was a trend towards better OS in the C3 subtype compared with C2, although the difference was not statistically significant (C2 vs. C3, HR = 1.48, 95% CI 0.92–2.40, p = 0.11, Fig. 3A). The heat map in Fig. 3B shows the expression of the 35 genes in different subtypes and their association with clinical features. In particular, subtype C1 was associated with a higher number of patients with mucinous adenocarcinoma (MAC, p < 0.01) and advanced TNM stages (p < 0.01, Table 1). Additionally, we assessed the correlation between the molecular subtypes related to hypoxia and lactate metabolism and recognized CMS. Our findings revealed that the C1 subtype had a higher proportion of CMS4 (68.48%), whereas CMS2 dominated the C3 subtype (61.01%, Fig. 3C). GSEA of subtypes C1 and C3 revealed that the hypoxia pathway was significantly upregulated in C1 (FDR = 0.02). In contrast, the oxidative phosphorylation pathway was significantly downregulated (FDR = 0.01, Fig. 3D), consistent with our expectations. In addition, C1 exhibited a significant upregulation of KRAS signaling, EMT, and angiogenesis (Fig. 3E). In contrast, C3 showed a significant upregulation of MYC targets v2, MYC targets v1, and E2F targets (Fig. 3F).

**Table 1:** Clinicopathological characteristics of the different molecular subtypes

	C1 (N = 92)	C2 (N = 324)	C3 (N = 159)	P value
Age				0.97
> 50	79 (85.87%)	281 (86.73%)	137 (86.16%)	
≤ 50	13 (14.13%)	43 (13.27%)	22 (13.84%)	
Sex				0.42
Female	43 (46.74%)	155 (47.84%)	66 (41.51%)	
Male	49 (53.26%)	169 (52.16%)	93 (58.49%)	
Tumor site				< 0.01
Right-sided colon	31 (33.70%)	142 (43.83%)	25 (15.72%)	
Left-sided colon	32 (34.78%)	89 (27.47%)	74 (46.54%)	
Rectum	12 (13.04%)	45 (13.89%)	26 (16.35%)	
Unknown	17 (18.48%)	48 (14.81%)	34 (21.38%)	
Pathological type				< 0.01
MAC	20 (21.74%)	52 (16.05%)	3 (1.89%)	
NMAC	72 (78.26%)	272 (83.95%)	156 (98.11%)	
T stage				< 0.01
T1	0 (0%)	11 (3.40%)	8 (5.03%)	
T2	7 (7.61%)	61 (18.83%)	35 (22.01%)	
T3	67 (72.83%)	217 (66.98%)	108 (67.92%)	
T4	18 (19.57%)	35 (10.80%)	8 (5.03%)	
N stage				< 0.01
N0	41 (44.57%)	200 (61.73%)	85 (53.46%)	
N1	25 (27.17%)	68 (20.99%)	51 (32.08%)	
N2	26 (28.26%)	56 (17.28%)	23 (14.47%)	
M stage				0.22
M0	63 (68.48%)	250 (77.16%)	124 (77.99%)	
M1	20 (21.74%)	41 (12.65%)	23 (14.47%)	
Mx	9 (9.78%)	33 (10.19%)	12 (7.55%)	
TNM stage				< 0.01
Stage I	5 (5.43%)	66 (20.37%)	34 (21.38%)	
Stage II	36 (39.13%)	128 (39.51%)	47 (29.56%)	
Stage III	31 (33.70%)	87 (26.85%)	55 (34.59%)	
Stage IV	20 (21.74%)	43 (13.27%)	23 (14.47%)	
Chemotherapy				0.51
Yes	41 (44.57%)	123 (37.96%)	61 (38.36%)	
No	51 (55.43%)	201 (62.04%)	98 (61.64%)	

MAC: mucinous adenocarcinoma; NMAC: non-mucinous adenocarcinoma

### Immune landscape of the different molecular subtypes

**Fig. 4:**
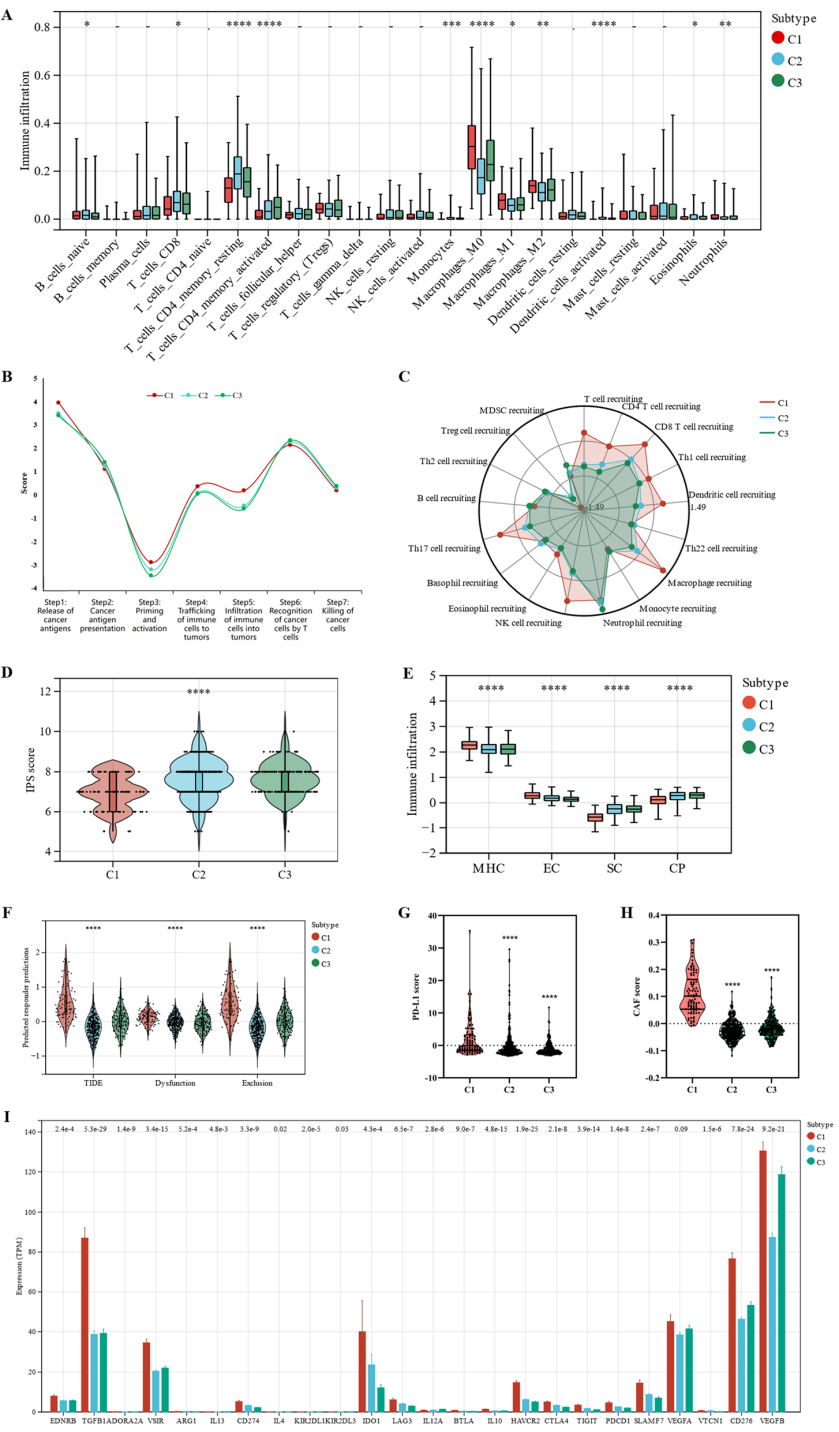
Immunoscape of the different molecular subtypes. **A** CIBERSORT revealed the infiltration of 22 types of immune cells in the different molecular subtypes. **B** Differences between the three subtypes in the cancer immune cycle. **C** Radar chart of the differences in the recruitment of immune cells by different molecular subtypes in the cancer immune cycle. **D** IPS of the three molecular subtypes. **E** Major histocompatibility complex (MHC) molecules, effector cells (EC), suppressor cells (SC), and checkpoint (CP) scores of the different molecular subtypes. **F** Total TIDE scores of the three molecular subtypes. **G** PD-L1 and **H** CAF score of three molecular subtypes. **I** Expression of 24 well-known immune checkpoint genes in the different subtypes. IPS: immunophenoscore; TIDE: Tumor Immune Dysfunction and Exclusion; CAF: cancer-associated fibroblast. *p < 0.05; **p < 0.01; ***p < 0.001; ****p < 0.001

As hypoxia is closely related to the immune microenvironment, we evaluated immune cell infiltration in the three subtypes. We analyzed the infiltration of 22 immune cells using CIBERSORT and found that the C1 subtype predominantly contained macrophages, including M1 (p = 0.02) and M2 (p < 0.01). Meanwhile, an increase in the number of CD8 + T cells was observed in subtypes C2 and C3 (p = 0.01). Resting CD4 + memory T cells (p < 0.01), activated CD4 + memory T cells (p < 0.01), and activated dendritic cells (p < 0.01) were significantly more abundant in subtypes C2 and C3 than in the C1 subtype (Fig. 4A).

The cancer immune cycle (CIC) comprises seven major steps necessary for the immune-mediated control of tumor growth, beginning with the release of antigens from cancer cells and ending with the killing of cancer cells. The three subtypes were also evaluated for the seven major steps of CIC. The C1 subtype was found to play a significant role in the release of cancer antigens, priming and activation, trafficking of immune cells to tumors, and infiltration of immune cells into tumors (Fig. 4B). Additionally, Fig. 4C suggests that immune cells such as T cells, macrophages, and NK cells were widely recruited in the C1 subtype. However, the performance of the C1 subtype was slightly lower than that of the C2 and C3 subtypes in the recognition of cancer cells by T cells and killing of cancer cells, which are key steps in antitumor immunity, despite its ability to enrich more immune cells.

To evaluate the immunocompetence status of the three subtypes, we assessed their IPS. The IPS of the C1 subtype was significantly lower than those of subtypes C2 and C3 (p < 0.01, Fig. 4D), indicating that the C1 subtype was less responsive to immunotherapy. Specifically, the C1 subtype exhibited higher scores for MHC molecules (p < 0.01) and effector cells (p < 0.01) and lower scores for suppressor cells (p < 0.01) and checkpoints (p < 0.01, Fig. 4E). These findings appear to be inconsistent with the poor prognosis associated with the C1 subtype. To further investigate this phenomenon, we analyzed the immune functions of the samples using the TIDE algorithm. The results showed that the C1 subtype had a significantly higher overall TIDE score (p < 0.01), tumor immune dysfunction score (p < 0.01), and tumor immune exclusion score (p < 0.01) than the C2 and C3 subtypes (Fig. 4F), suggesting that immune escape was more common in the C1 subtype. Additionally, the PD-L1 score (p < 0.01, Fig. 4G) and cancer-associated fibroblast (CAF) score (p < 0.01, Fig. 4H) were significantly higher in the C1 subtype than in subtypes C2 and C3. We also evaluated the expression of 24 well-known immune checkpoint genes across the different subtypes. The results showed that these molecules, including CD274 (PD-L1), LAG3, CTLA4, and CD276, were upregulated in the C1 subtype (Fig. 4I).

### Mutation mapping of the different molecular subtypes

**Fig. 5:**
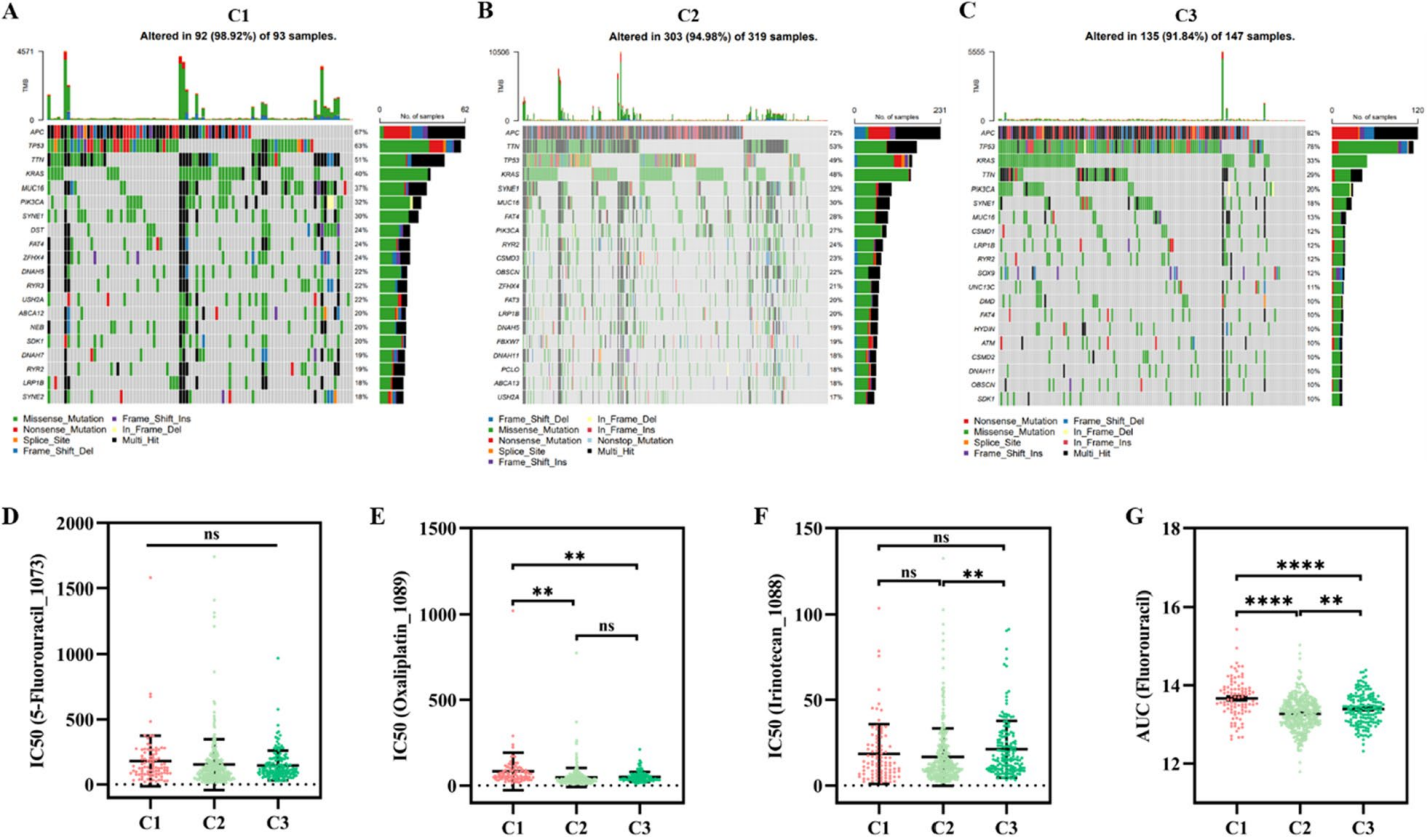
Mutation mapping and drug sensitivity. **A**–**C** TMB, mutation frequency and types in the three molecular subtypes. Comparison of the IC_50_ values of various chemotherapies in the three subtypes, including **D** 5-fluorouracil, **E** oxaliplatin, and **F** irinotecan. **G** Comparison of the AUC values of fluorouracil in the three subtypes based on the CTRP2 database. IC_50_: half maximal inhibitory concentration; AUC: area under the curve; TMB: tumor mutation burden

To better understand the distinctions between the various subtypes, we examined them at the gene mutation level. Subtypes C1 and C2 exhibited a high total mutation burden (TMB), whereas subtype C3 exhibited a low TMB (p < 0.01, Fig. 5A–C). The top 20 genes in terms of mutation frequency were analyzed for each subtype. *APC* showed increasing mutation frequency in subtypes C1, C2, and C3 (67% vs. 72% vs. 82%), whereas *MUC16* (37% vs. 30% vs. 13%) and *PIK3CA* (32% vs. 27% vs. 20%) showed decreasing mutation frequency in subtypes C1, C2, and C3, respectively. Additionally, *TP53* mutation frequency was the highest in the C3 subtype (78%), followed by C1 (63%) and C2 (49%). The C2 subtype had the highest *KRAS* mutation frequency (48%), followed by subtypes C1 (40%) and C3 (33%).

### Drug sensitivity

In clinical practice, stage II CRC patients with high-risk factors and patients with stage III and IV CRC require postoperative chemotherapy. Sensitivity to commonly used chemotherapeutic agents greatly affects the outcomes and prognosis of patients with CRC. Therefore, we predicted the sensitivity of patients with the three subtypes to the most commonly used chemotherapeutic agents for CRC, namely 5-FU, oxaliplatin, and irinotecan, based on the GDSC2 and CTRP2 databases. The sensitivities of the C1, C2, and C3 subtypes to 5-FU did not differ significantly in the GDSC2 database (p = 0.51, Fig. 5D). However, patients in the C1 subtype had a significantly higher half-maximal inhibitory concentration (IC_50_) for oxaliplatin than those in subtypes C2 (p < 0.01) and C3 (p < 0.01); no significant difference in oxaliplatin sensitivity was found between patients in subtypes C2 and C3 (p = 0.45, Fig. 5E). There was no significant difference in the IC_50_ for irinotecan between subtypes C1 and C2 (p = 0.39) or C3 (p = 0.22). Patients with the C3 subtype had slightly higher IC_50_ values than those with the C2 subtype (p < 0.01, Fig. 5F). When we switched to the CTRP2 database, we found that the AUC for 5-FU was higher in the C1 subtype than in the C2 (p < 0.01) and C3 (p < 0.01, Fig. 5G) subtypes, suggesting that the C1 subtype was the least sensitive to 5-FU. In addition, ssGSEA found higher stemness enrichment scores in C1 (Figure S2A) and stemness-associated genes such as LGR5 and CD34 were significantly overexpressed in the C1 subtype than in the other two subtypes (Figure S2B, C). The differences in characteristics of stemness may explain the differences in drug sensitivity between the subgroups.

**Fig. 6:**
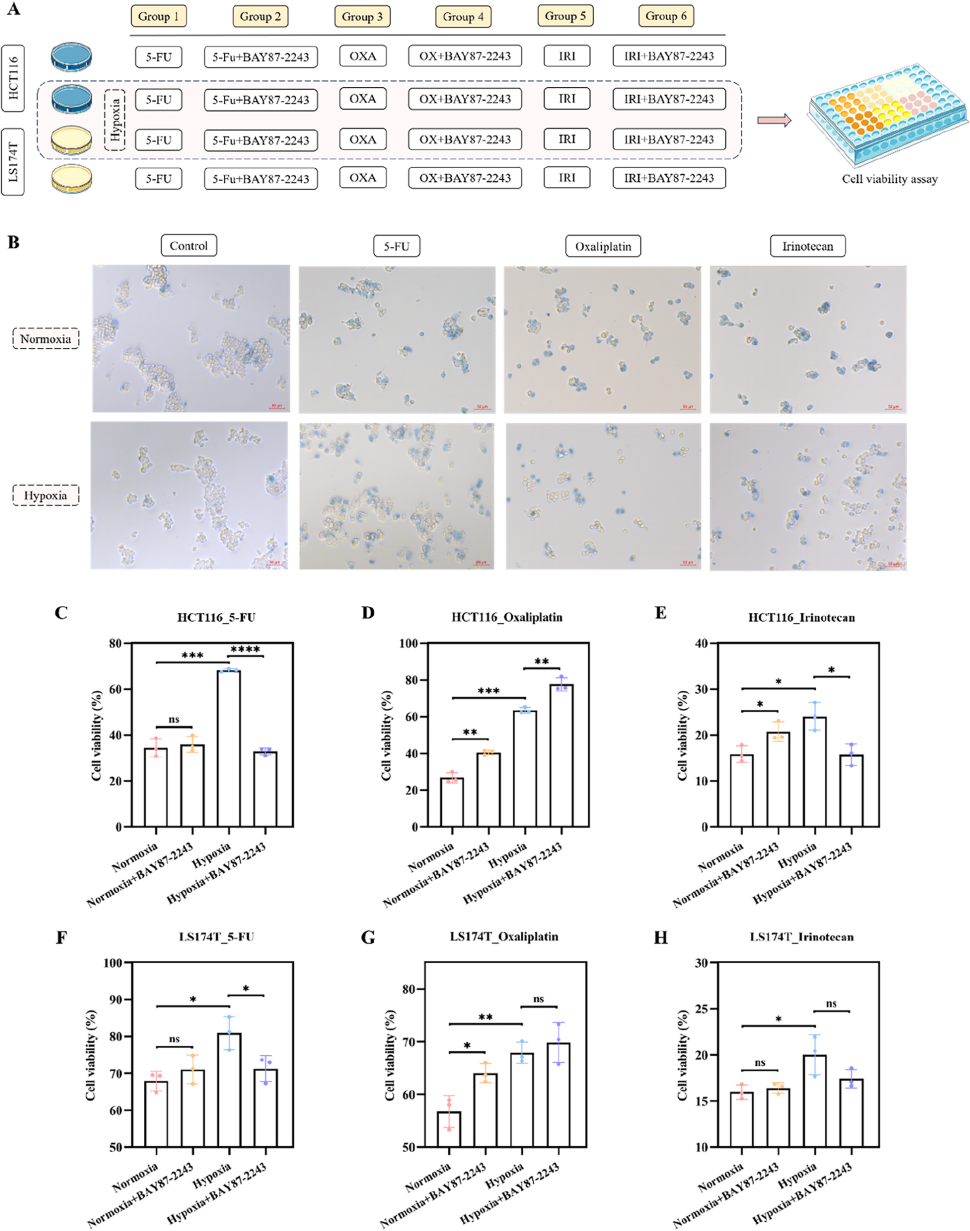
In vitro cell viability assay under normoxic and hypoxic circumstances. **A** The experimental workflow. **B** Trypan blue staining of cells subjected to different oxygen concentrations and treated with different chemotherapy drugs. CCK8 experiments after normoxia or hypoxia and **C** 5-FU, **D** oxaliplatin, or **E** irinotecan treatment combined with or without BAY87-2243 in HCT116 cells. CCK8 experiments after normoxia or hypoxia and **F** 5-FU, **G** oxaliplatin, or **H** irinotecan treatment combined with or without BAY87-2243 in LS174T cells. OXA: oxaliplatin; IRI: irinotecan; CCK8: Cell Counting Kit-8; ns: no significance. *p < 0.05; **p < 0.01; ***p < 0.001; ****p < 0.001

To gain a better understanding of the sensitivity of tumor cells to chemotherapeutic drugs under hypoxic conditions, we performed in vitro experiments under hypoxic (1% O_2_) and normoxic (21% O_2_) conditions. The experimental workflow is shown in Fig. 6A. The sensitivity of the CRC cell lines HCT116 and LS174T to the conventional chemotherapeutic agents 5-FU, oxaliplatin, and irinotecan was characterized. Moreover, the cells were treated with or without the HIF-1 inhibitor BAY87-2243 under hypoxic and normoxic conditions. Trypan blue staining revealed that the survival rate of tumor cells was higher after 48 h of hypoxia compared with that under normoxic conditions at the same concentrations of 5-FU (7.5 µM), oxaliplatin (7.5 µM), or irinotecan (10 µM in HCT116 cells and 50 µM in LS174T cells) (Fig. 6B). These results were confirmed by CCK8 experiments (Fig. 6C–H). Notably, under hypoxic conditions, BAY87-2243 increased the sensitivity of HCT116 and LS174T cells to 5-FU to normoxic levels (Fig. 6C, F). However, under both normoxic and hypoxic conditions, BAY87-2243 decreased the sensitivity of HCT116 cells to oxaliplatin (Fig. 6D); this was also observed in LS174T cells under normoxic conditions (Fig. 6G). Under hypoxic conditions, BAY87-2243 increased the sensitivity of HCT116 and LS174T cells to irinotecan (Fig. 6E, H).

### Construction of a prognostic prediction model

**Fig. 7:**
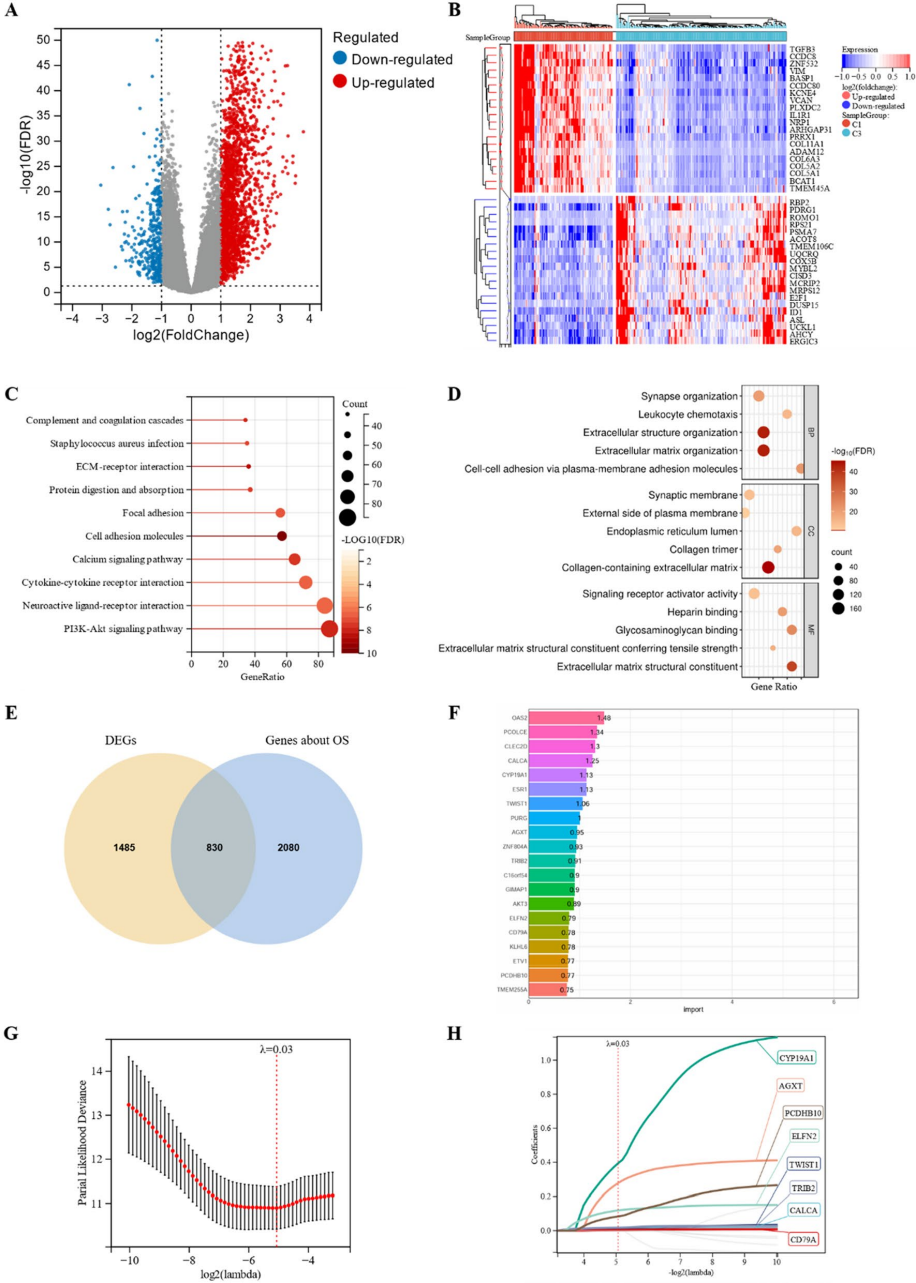
Screening process for the characterized genes. **A** Volcano plot of the DEGs between subtypes C1 and C3. **B** Heatmap of the top 20 upregulated and downregulated genes. **C** Top 10 KEGG pathways of the DEGs. **D** Top 5 pathways of the DEGs involved in GO BP, CC, and MF terms, respectively. **E** Venn diagram of the DEGs between subtypes C1 and C3 and genes associated with OS. **F** The top 20 characterized genes screened using the random forest model. **G**, **H** The LASSO regression analysis and partial likelihood deviance on the prognostic genes selected by the random forest model. DEGs: differentially expressed genes; BP: biological process; CC: cellular component; MF: molecular function; OS: overall survival; LASSO: least absolute shrinkage and selection operator

To clarify the molecular mechanisms underlying hypoxic tumors and their impact on the OS of patients with CRC, we compared the C1 subtype, which had the worst prognosis, with the C3 subtype, which had the best prognosis. Differential expression analysis using DESeq2 revealed that 1953 genes were upregulated in the C1 subtype, while 362 genes were downregulated (Fig. 7A). The heatmap in Fig. 7B displays the top 20 genes that were upregulated and downregulated. KEGG analysis of these 2315 DEGs revealed that they were predominantly enriched in cell adhesion molecules, ECM-receptor interactions, and the PI3K-Akt signaling pathway (Fig. 7C). GO functional enrichment analysis revealed that these DEGs were significantly enriched in extracellular structure organization, extracellular matrix organization, collagen-containing extracellular matrix, and extracellular matrix structural components, suggesting a close association between DEGs and extracellular matrix (Fig. 7D).

**Fig. 8:**
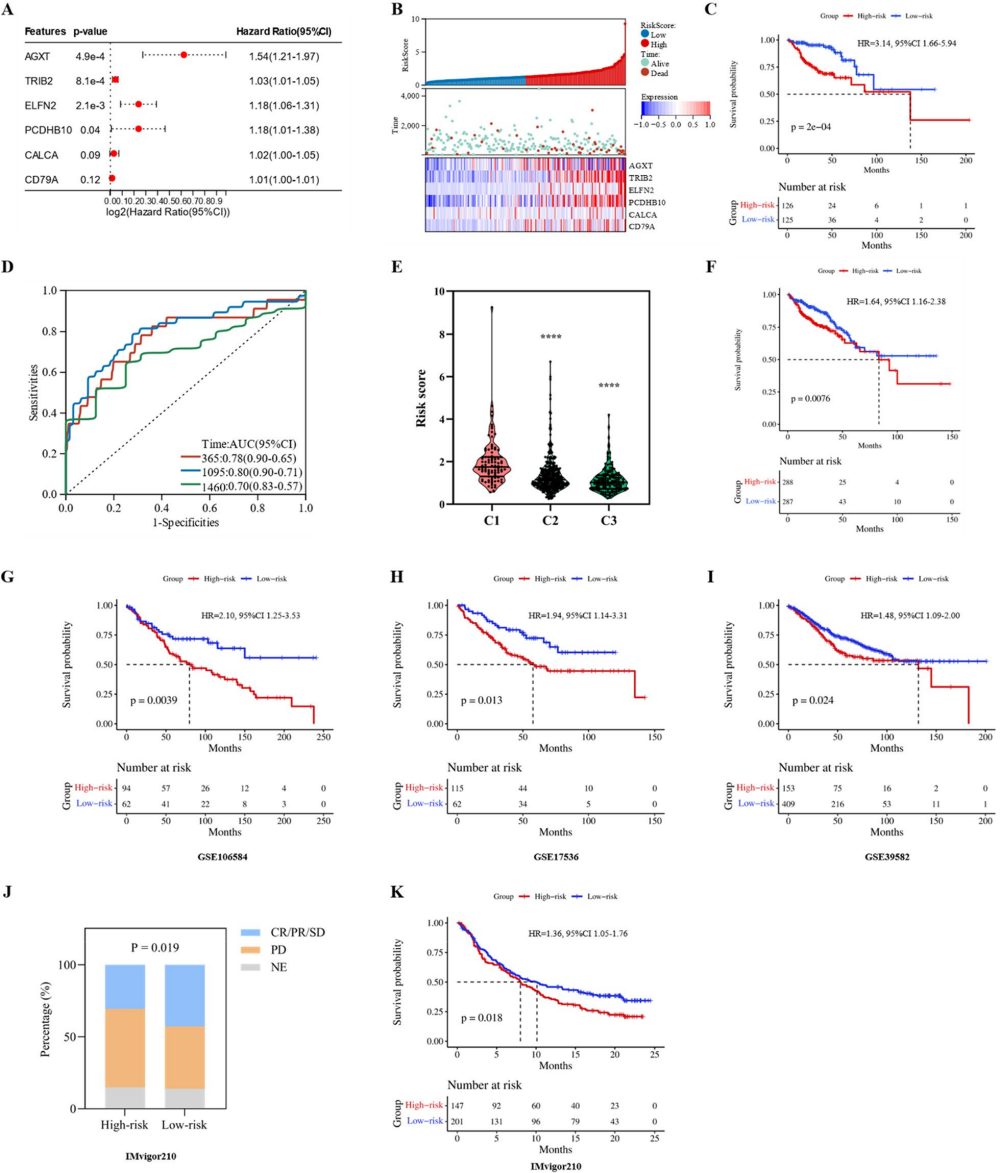
Construction and validation of the HLM score. **A** Multivariate Cox regression analysis to construct the prognostic predictive model. **B** Distribution of risk scores, clinical events, and the expression of model genes in patients under subtypes C1 and C3. **C** The Kaplan–Meier survival curve for the low-risk and high-risk groups of patients in subtypes C1 and C3. **D** Time-dependent ROC curves for patients in subtypes C1 and C3. **E** Risk score distribution in subtypes C1, C2, and C3. The Kaplan–Meier survival curve for the low-risk and high-risk groups in **F** the total TCGA cohort and the **G** GSE106584, **H** GSE17536, and **I** GSE39582 datasets. **J** Proportion of patients who respond to anti-PD-1/L1 immunotherapy with high or low risk scores in the IMvigor210 cohort. **K** Kaplan–Meier survival curve for the low-risk and high-risk groups in the IMvigor210 cohort. CR: complete response; PR: partial response; SD: stable disease; PD: progressive disease; NE: not evaluated. ****p < 0.0001

Univariate Cox regression analysis was performed to identify 2910 OS-related genes. After determining their intersection with the obtained 2315 DEGs, 830 OS-related DEGs were identified (Fig. 7E). Subsequently, the 830 DEGs were screened using a random forest model, and the top 20 genes were selected (Fig. 7F). To avoid problems with covariance and overfitting, we further screened these 20 DEGs using the LASSO method, which identified eight potential genes (Fig. 7G, H). Finally, we conducted a multivariate Cox regression analysis based on these eight genes and obtained six DEGs to construct a prognostic prediction signature, which we named the HLM score (Fig. 8A).

### Validation of the predictive efficacy of the HLM score

We divided the 251 patients in the C1 and C3 subtypes into high-risk and low-risk groups based on their HLM scores. A marked decrease in the OS of CRC patients was observed as the HLM score increased (Fig. 8B). The Kaplan–Meier curves indicated that patients in the high-risk group had significantly worse OS (HR = 3.14, 95% CI 1.66–5.94, p < 0.01, Fig. 8C). The AUCs of the ROC for predicting 1-, 3-, and 4-year OS were 0.78 (95% CI 0.90–0.651), 0.80 (95% CI 0.90–0.71), and 0.70 (95% CI 0.83–0.57), respectively, suggesting superior predictive efficacy (Fig. 8D). Additionally, compared with clinical factors alone, we found that when we combined the HLM score with clinical factors such as age and T, N, and M stage, it significantly improved the AUCs (Figure S3). When the HLM score was applied to the entire TCGA CRC cohort (n = 575), the C1 subtype had the highest HLM score, the C3 subtype had the lowest HLM score, and the C2 subtype had an intermediate HLM score, consistent with our previous molecular subtyping results (Fig. 8E). The TCGA CRC cohort was also divided into high-risk and low-risk groups based on the HLM scores. GSEA of the high-risk and low-risk groups revealed that the hypoxia and angiogenesis pathway were significantly upregulated in the high-risk group. In contrast, the oxidative phosphorylation pathway was significantly downregulated (Figure S4A). The expression of HIF-1α and lactate metabolism-related genes such as IGFBP3, TGFB3, and CAV1 were significantly higher in the high-risk group than in the low-risk group (Figure S4B, D). Patients in the high-risk group had a significantly worse OS (HR = 1.64, 95% CI 1.16–2.38, p < 0.01, Fig. 8F). In addition, the high-risk group, similar to the C1 subtype, exhibited a higher proportion of CMS4 while the low-risk group exhibited a higher proportion of CMS2 (Figure S5A). The effect of CMS alone on CRC prognostic stratification was insignificant. However, the HLM score in concert with CMS demonstrated the capacity to refine CRC prognostic stratification (Figure S5B, C).

To validate the efficacy of the HLM score more extensively, we obtained three datasets from the GEO database. These datasets were categorized into high- and low-risk groups based on the HLM scores. Results showed that in the GSE106584 (HR = 2.10, 95% CI 1.25–3.53, p < 0.01, Fig. 8G), GSE17536 (HR = 1.94, 95% CI 1.14–3.31, p = 0.01, Fig. 8H), and GSE39582 (HR = 1.48, 95% CI 1.09–2.00, p = 0.02, Fig. 8I) datasets, patients in the high-risk group displayed inferior OS than patients in the low-risk group. Additionally, we analyzed the IMvigor210 immunotherapy cohort, in which patients in the high-risk group had a higher proportion of progressive disease (54.42% vs. 43.48%) and fewer patients with complete response/partial response/stable disease (30.61% vs. 40.79%, p = 0.02; Fig. 8J) than the low-risk group. The Kaplan–Meier curve also suggested worse OS for patients in the high-risk group in the IMvigor210 cohort than in the low-risk group (HR = 1.36, 95% CI 1.05–1.76, p = 0.02, Fig. 8K). Besides, we assessed the efficacy of the HLM score in these four validation sets through ROC curves, risk heatmaps, and calibration curves, all of which suggest that the HLM score is robust (Figure S6).

### ssGSEA and scRNA-seq analysis to assess immune cell infiltration

**Fig. 9:**
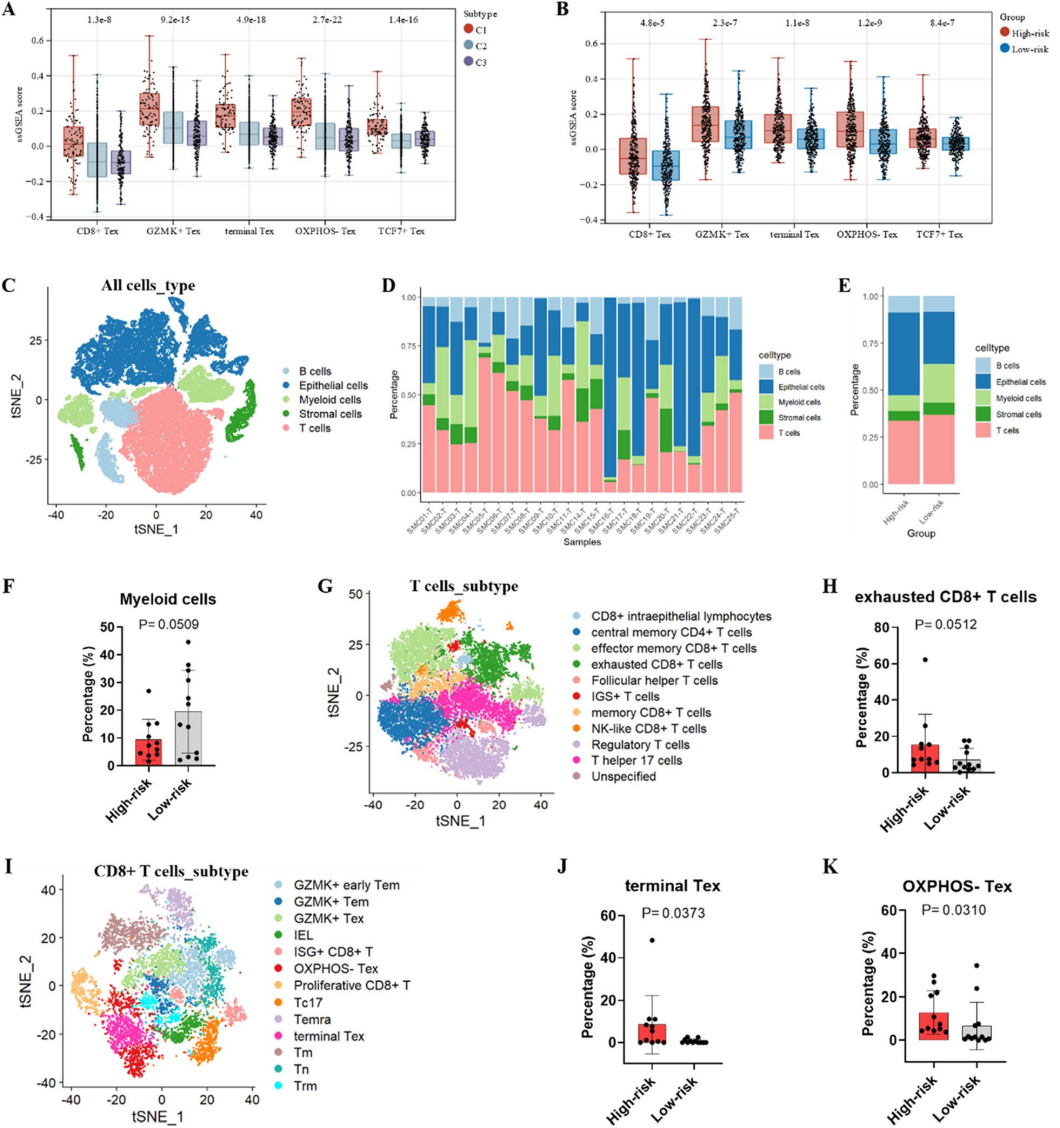
ssGESA and scRNA-seq analysis to assess immune cell infiltration. Enrichment scores of CD8 + Tex, GZMK + Tex, terminal Tex, OXPHOS- Tex, and TCF7 + Tex in (**A**) different molecular subtypes and **B** the high-risk and low-risk groups based on the HLM score in the TCGA cohort. **C** t-SNE plot of 47,285 cells from 23 patients with CRC in the GSE132465 cohort. **D** Percentage of the five major cell types in each sample. **E** Percentage of the five major cell types in the high-risk and low-risk groups based on the HLM score. **F** Percentage of myeloid cells in the high-risk and low-risk groups. **G** t-SNE plot of T cells in the GSE132465 cohort. **H** Percentage of CD8 + Tex in the high-risk and low-risk groups. **I** t-SNE plot of CD8 + T cells in the GSE132465 cohort. Percentage of **J** terminal Tex and **K** OXPHOS- Tex in the high-risk and low-risk groups. Tex: exhausted T cells; t-SNE: t-distributed stochastic neighbor embedding

The ssGSEA results indicated that the enrichment scores for CD8 + Tex, GZMK + Tex, terminal Tex, OXPHOS- Tex, and TCF7 + Tex were significantly higher in the C1 subtype than in subtypes C2 and C3 (p < 0.01, Fig. 9A). Similar results were obtained when TCGA CRC samples were categorized into high- and low-risk groups based on the HLM score (p < 0.01, Fig. 9B). To further validate these findings, publicly available scRNA-seq data were analyzed. After selection and filtering, 47,285 cells were included in the analysis. These cells clustered into five major types: B cells, T cells, myeloid cells, stromal cells, and epithelial cells (Fig. 9C, D). Based on the HLM score of each sample after pseudo-bulk analysis, the 23 CRC samples from the scRNA-seq data source were categorized into the high-risk (n = 11) and low-risk (n = 12) groups. The proportion of myeloid cells was slightly higher in the low-risk group than in the high-risk group (p = 0.05, Fig. 9E, F). We then performed subcluster annotation on 16,065T cells from these CRC samples (Fig. 9G). The proportion of exhausted CD8 + T cells (p = 0.05, Fig. 9H) and CD8 + intraepithelial lymphocytes (p = 0.04, Figure S7A) was higher in the high-risk group than in the low-risk group. To determine specific differences in the proportion of CD8 + T cells between the two groups, we performed further subpopulation annotation of CD8 + T cells (Fig. 9I). The results showed that the proportions of terminal Tex (p < 0.05, Fig. 9J), OXPHOS-Tex (p < 0.05, Fig. 9K) and GZMK + early Tem (effective memory T cells) (p < 0.05, Figure S7B) were significantly higher in the high-risk group than in the low-risk group. These findings suggest that HRGs and LMRGs may play a role in CD8 + T cell exhaustion in CRC.

In addition, we classified the 23 samples of scRNA-seq into C1/C2/C3 subtypes. It was found that, similar to the HLM score classification results, for the T cell subpopulation, exhausted CD8 + T cells especially OXPHOS- Tex were significantly higher in C1 than in C2 and C3 subtypes (Figure S7C, D). Furthermore, we found that naïve T (Tn) cells were lower in C1 than in C2, C3 subtypes (Figure S7D). Tn cells are precursors of effector and memory T cell subsets. Differences in Tn proportion between the three subtypes may be attributed to the fact that the proportion of exhausted T cells varies between the subtypes.

### Establishment of a predictive nomogram

**Fig. 10:**
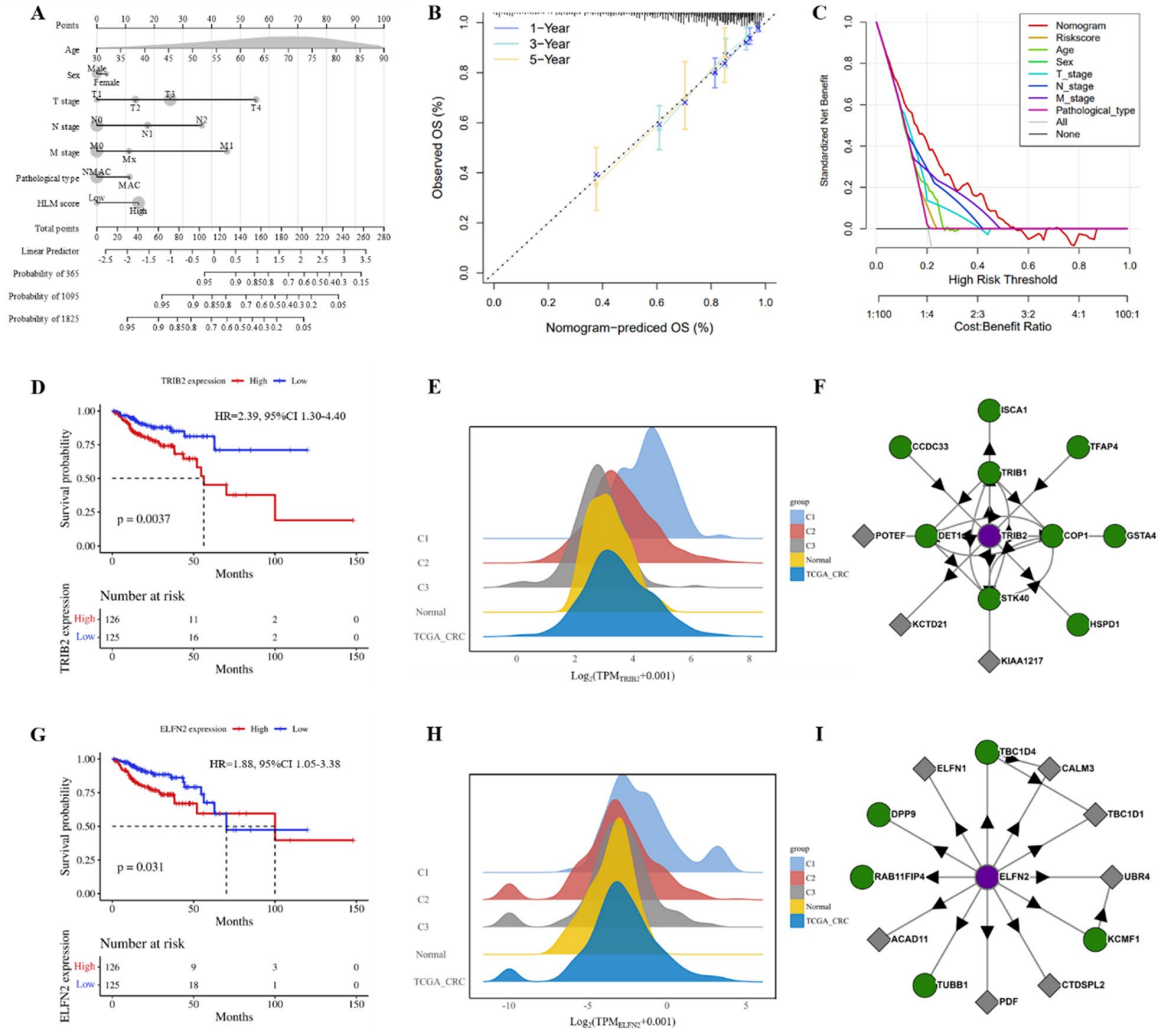
Establishment of the prognostic predictive nomogram and single gene analysis. **A** Nomogram for predicting 1-, 3-, and 4-year OS using the HLM score and other clinical features. **B** Calibration plot showing the differences between nomogram-predicted OS and observed OS. **C** DCA demonstrating the net clinical benefits associated with the nomogram. **D** Kaplan–Meier survival curve for the high-expression and low-expression groups depending on TRIB2 expression. **E** TRIB2 expression in normal tissues, different molecular subtypes, and the TCGA CRC cohort. **F** Proteins that interact with TRIB2. **G** Kaplan–Meier survival curve for the high-expression and low-expression groups depending on ELFN2 expression. **H** ELFN2 expression in normal tissues, different molecular subtypes, and the TCGA CRC cohort. **I** Proteins that interact with ELFN2. DCA: decision curve analysis; MAC: mucinous adenocarcinoma; NMAC: non-mucinous adenocarcinoma; OS: overall survival; CRC: colorectal cancer

A predictive nomogram based on seven factors, namely age, sex, T stage, N stage, M stage, pathological type, and HLM score, was constructed to visualize the prognosis of patients in the total TCGA CRC cohort. The nomogram could predict the OS of patients with CRC with a C-index of 0.78 (Fig. 10A). Calibration curves for the nomogram also showed ideal prediction accuracy (Fig. 10B). The DCA curve demonstrated that the nomogram provided more net clinical benefits than the clinical characteristics alone (Fig. 10C). The nomogram based on the HLM score could predict both short- and long-term OS in patients with CRC and assist their clinical management.

### Single gene analysis

To understand how the HLM score predicts the prognosis of patients with CRC, we performed a single gene analysis of six characterized genes in C1 and C3 subtypes, and the Kaplan–Meier curve indicated that the overexpression of TRIB2 or ELFN2 predicted poorer OS in patients with CRC, with HRs of 2.39 (95% CI 1.30–4.40, p < 0.01, Fig. 10D) and 1.88 (95% CI 1.05–3.38, p = 0.03, Fig. 10G), respectively. The expression matrix revealed that TRIB2 expression was significantly higher in CRC tissues than in normal tissues. In CRC, the expression of TRIB2 increased sequentially in subtypes C3, C2, and C1 (p < 0.01, Fig. 10E). Additionally, ELFN2 was highly expressed in CRC, particularly in the C1 subtype (p < 0.01, Fig. 10H). However, TRIB2 and ELFN2 are not included in the existing set of HRGs and LMRGs. To obtain information on the proteins interacting with TRIB2 and ELFN2, we consulted the BioPlex Interactome database. Our findings indicated that ISCA1, which interacts with TRIB2, is closely related to lactate metabolism (Fig. 10F). Similarly, KLHL24, which interacts with ELFN2, is closely associated with hypoxia (Fig. 10I). Therefore, we infer that TRIB2 and ELFN2 are associated with hypoxia and lactate metabolism.

## Discussion

Hypoxia and acidosis within the TME, caused by the increased secretion of lactic acid and H^+^ as end products of glycolysis [49], are markers of solid tumors, including CRC. These conditions determine the selection of invasive and aggressive malignant clones that display resistance to radiotherapy, conventional chemotherapy, targeted therapy, or immunotherapy [50]. Due to the extensive intratumor heterogeneity and complex genetic and biological characteristics of CRC [51], predicting the outcome of patients with CRC remains challenging. To address this, we performed molecular subtyping of CRC based on HRGs and LMRGs and developed an HLM scoring system to predict prognosis and treatment response in patients with CRC.

Currently, molecular subtyping of CRC has shifted from a mutation-centered to a transcriptome-centered approach and from supervised to unsupervised clustering [52]. For instance, the CMS approach proposed by Guinney et al. in 2015, which is based on transcriptome analysis, has gained widespread acceptance [38]. To the best of our knowledge, this is the first study to propose an unsupervised clustering method for the molecular subtyping of CRC, based on HRGs and LMRGs. Our molecular subtyping method classified CRC into three subtypes and distinguished between their prognostic differences. In addition, the molecular subtypes generated correlated with the CMS and can complement it to guide the individualized treatment of patients with CRC.

Subsets of immune cells form a complicated network and cross-talk in different ways within the tumor [53]. Rather than their mere presence, the type of immune cells is more likely to be crucial. The C1 subtype predominantly contains macrophages, including M1 and M2 macrophages. Through specific differentiation, macrophages can evolve into two different polarization states in CRC: classically activated M1 (pro-inflammatory) and M2 (anti-inflammatory) macrophages. CRC originates from the epithelium; hence, TAMs are predominantly pro-inflammatory M1 macrophages in the early stages. However, they tend to convert to the anti-inflammatory and cancer-promoting M2 phenotype with tumorigenic activity as tumor cells utilize them to support the growth and progression of advanced CRC [54].

The results indicate that the hypoxic C1 subtype has abundant immune cell infiltration. However, the ability of these immune cells to kill tumor cells is inferior to that of the other two subtypes, suggesting that the number of infiltrating immune cells and the distribution of immune cell subsets play an important role in influencing antitumor immunity in the TME. TIDE analysis similarly demonstrated this, with the C1 subtype exhibiting higher tumor immune dysfunction and tumor immune exclusion scores than the other subtypes. This suggests that patients with the hypoxic subtype have a higher prevalence of immune escape from the TME, as evidenced by the upregulation of 24 well-known immune checkpoint genes. Additionally, the C1 subtype had a significantly higher CAF score than the C2 and C3 subtypes. CAFs play a crucial role in the reactive stroma of the TME and significantly influence tumor biology, including angiogenesis, invasion, immune evasion, metastasis, and drug resistance [55, 56]. Hypoxia activates resident fibroblasts by activating the reactive oxygen species (ROS) and HIF-1α pathways, transforming them into CAFs. CAFs undergo metabolic reprogramming to adapt to the TME and support glycolysis under hypoxic conditions. Increased glycolysis contributes to tumor cell proliferation [57, 58]. Furthermore, hypoxia affects chemokine and cytokine production by CAFs, leading to an increase in the proportion of immune cells associated with an immunosuppressive microenvironment [59].

Our study developed the HLM scoring system to predict the OS and efficacy of immunotherapy in patients with CRC using univariate Cox regression, the random forest model, LASSO, and multivariate Cox regression analyses. Notably, the group with a high HLM score exhibited a high proportion of CD8 + Tex infiltration, especially terminal Tex and OXPHOS CD8 + T cells. CD8 + T-cells are critical for eliminating cancer cells. We observed a reduction in both the number and function of CD8 + T cells in patients with high levels of hypoxia. In cancer, the continuous stimulation of antigen receptors can lead to an alternative differentiation trajectory known as T cell exhaustion, when antigens cannot be completely eliminated [60]. In CD8 + T cells, sustained exposure to hypoxia rapidly accelerates their differentiation to terminal exhaustion and represses antitumor immunity [61]. T cell exhaustion can be classified into four stages: T cell exhaustion progenitors 1, T cell exhaustion progenitors 2, T cell exhaustion intermediate and T cell exhaustion terminally [62]. Hypoxia results in abnormal OXPHOS in the mitochondria, which hinders T cell proliferation and promotes the transcriptional and metabolic reprogramming of the progenitor Tex (Tpex) and their differentiation into terminal Tex [63, 64]. Monoclonal antibody-based immunotherapies, such as anti-PD-L1/PD-1 agents, can become ineffective when T cells reach a stage of terminal exhaustion [62]. This explains why we observed that the hypoxic C1 subtype highly expresses numerous immune checkpoint molecules but still suffers from poor immunotherapeutic outcomes.

The HLM score was constructed based on six genes, *AGXT*, *TRIB2*, *ELFN2*, *PCDHB10*, *CALCA*, and *CD79A*, which have been previously reported to be associated with the tumorigenesis and progression of various cancer types, including CRC, as well as chemotherapy resistance [65–69]. When considering individual genes, we discovered that the high expression of TRIB2 and ELFN2 was associated with poor prognosis in patients with CRC. TRIB2 is a specialized member of the tribbles pseudokinase family that interacts with MAPK, AP4, CDC25, OCT3/4, C/EBP-alpha, ubiquitin E3 ligases, PCBP2, and AKT to regulate cellular processes, such as the cell cycle, senescence, stem cell pluripotency, protein degradation, and cell survival [70]. One study discovered that TRIB2 impedes tumor cell senescence, stimulates proliferation, and triggers cell cycle arrest via AP4/p21 signaling in CRC [66]. *ELFN2* is a newly discovered hypomethylated gene that can inhibit the formation of protein phosphatase complexes and suppress the activity of protein phosphatase 1 by regulating it [71]. ELFN2 is also involved in glioblastoma cell autophagy [71], pancreatic cancer radiotherapy resistance [72], gastric cancer invasion [67], and endometrial cancer progression [73].

However, this study has certain limitations. First, most of our analyses and conclusions are based on data from public databases. Although our findings were validated across multiple datasets, it is important to acknowledge the possibility of bias. Moreover, we did not delve into the mechanisms by which hypoxia causes insensitivity to chemotherapeutic agents in patients with CRC. More in-depth in vitro and in vivo experiments are required to further explore the mechanisms underlying hypoxia-induced resistance to chemotherapy.

## Conclusions

In conclusion, we molecularly typed patients with CRC based on their HRGs and LMRGs and revealed the immunologic and genetic characteristics of the different molecular subtypes. In addition, we constructed an HLM score that can be used to predict the prognosis and efficacy of immunotherapy of patients with CRC. This score has been validated in multiple datasets and has the potential to guide individualized and precise diagnosis and treatment of patients with CRC.

### Supplementary Information

Supplementary Material 1. Figure S1. Hypoxia- and lactate metabolism-related microenvironments of the three subtypes. A Hypoxia microenvironment. B Lactate metabolism-related microenvironment. Figure S2. Stemness enrichment scores and expression of stemness-associated genes in different subtypes. A Stemness enrichment scores. B Exprission of LGR5. C Exprission of CD34. Figure S3. Time-dependent ROC curves for predicting 1-, 3-, and 5-year OS. A Clinical factors alone. B The HLM score combined with clinical factors. Figure S4. Hypoxia- and lactate metabolism-related microenvironments of the high-risk and low-risk groups. A GSEA of the high-risk and low-risk groups. B Hypoxia- and lactate metabolism-related microenvironment of the high-risk and low-risk groups. Figure S5. Comparison of HLM Score and CMS. A Distribution of the CMS in the high-risk and low-risk group. B CMS for CRC prognostic stratification. C HLM score combined with CMS for CRC prognostic stratification. Figure S6. ROC curves, risk heatmaps, and calibration curves in the validation sets. A GSE106584, B GSE17536, C GSE39582 and D IMvigor210. Figure S7. scRNA-seq analysis to assess immune cell infiltration. A Percentage of T cell subpopulation infiltration in high-risk vs. low-risk groups. B Percentage of CD8+ T cell subpopulation infiltration in high-risk vs. low-risk groups. C Percentage of T cell subpopulation infiltration in different molecular subtypes. D Percentage of CD8+ T cell subpopulation infiltration in different molecular subtypes. Tem, effective memory T cells; IEL, intraepithelial lymphocyte; Tc17, IL-17-producing CD8+ T cells; Tm, memory T cells; Tn, naïve T cells; Trm, tissue-resident memory T cells.

Supplementary Material 2.

## Data Availability

Publicly available datasets were analyzed in this study. These data can be found in the TCGA (https://portal.gdc.cancer.gov/), GEO (https://www.ncbi.nlm.nih.gov/), and IMvigor210CoreBiologies (http://research-pub.gene.com/IMvigor210CoreBiologies) databases.

## Abbreviations

AUC: Area under the curve
CAF: Cancer-associated fibroblast
CIC: Cancer immune cycle
CMS: Consensus molecular subtypes
CTRP: Cancer Therapeutics Response Portal
DCA: Decision curve analysis
DEG: Differentially expressed gene
EC: Effector cell
EMT: Epithelial-mesenchymal transition
GDSC: Genomics of drug sensitivity in cancer
GO: Gene ontology
GSEA: Gene set enrichment analysis
HIF: Hypoxia-inducible factor
HRG: Hypoxia-related gene
KEGG: Kyoto Encyclopedia of Genes and Genomes
LASSO: Least absolute shrinkage and selection operator
LMRG: Lactate metabolism-related genes
MAF: Mutation annotation format
MHC: Major histocompatibility complex
OS: Overall survival
ROC: Receiver operating characteristic
ROS: Reactive oxygen species
SC: Suppressor cells
scRNA-seq: Single-cell RNA sequencing
TAM: Tumor-associated macrophage
TCIA: The cancer immunome atlas
Tem: Effective memory T cells
Tex: Exhausted T cells
TIDE: Tumor immune dysfunction and exclusion
TMB: Tumor mutation burden
TPM: Transcripts per million
UMI: Unique molecular identifier
