# Supplementary material for: Novel hypoxia- and lactate metabolism-related molecular subtyping and prognostic signature for colorectal cancer
Source: J Transl Med. 2024 Jun 20;22:587. doi: 10.1186/s12967-024-05391-5 (PMC11191174; doi:10.1186/s12967-024-05391-5)
Supplement: Supplementary file 1 — Supplementary Material 1. Figure S1. Hypoxia- and lactate metabolism-related microenvironments of the three subtypes. A Hypoxia microenvironment. B Lactate metabolism-related microenvironment. Figure S2. Stemness enrichment scores and expression of stemness-associated genes in different subtypes. A Stemness enrichment scores. B Exprission of LGR5. C Exprission of CD34. Figure S3. Time-dependent ROC curves for predicting 1-, 3-, and 5-year OS. A Clinical factors alone. B The HLM score combined with clinical factors. Figure S4. Hypoxia- and lactate metabolism-related microenvironments of the high-risk and low-risk groups. A GSEA of the high-risk and low-risk groups. B Hypoxia- and lactate metabolism-related microenvironment of the high-risk and low-risk groups. Figure S5. Comparison of HLM Score and CMS. A Distribution of the CMS in the high-risk and low-risk group. B CMS for CRC prognostic stratification. C HLM score combined with CMS for CRC prognostic stratification. Figure S6. ROC curves, risk heatmaps, and calibration curves in the validation sets. A GSE106584, B GSE17536, C GSE39582 and D IMvigor210. Figure S7. scRNA-seq analysis to assess immune cell infiltration. A Percentage of T cell subpopulation infiltration in high-risk vs. low-risk groups. B Percentage of CD8+ T cell subpopulation infiltration in high-risk vs. low-risk groups. C Percentage of T cell subpopulation infiltration in different molecular subtypes. D Percentage of CD8+ T cell subpopulation infiltration in different molecular subtypes. Tem, effective memory T cells; IEL, intraepithelial lymphocyte; Tc17, IL-17-producing CD8+ T cells; Tm, memory T cells; Tn, naïve T cells; Trm, tissue-resident memory T cells. [file 12967_2024_5391_MOESM1_ESM.docx]

**Supplementary figures:**

**Figure S1.** Hypoxia- and lactate metabolism-related microenvironments of the three subtypes.

**Figure S2.** Stemness enrichment scores and expression of stemness-associated genes in different subtypes.

**Figure S3.** Time-dependent ROC curves for predicting 1-, 3-, and 5-year OS.

**Figure S4.** Hypoxia- and lactate metabolism-related microenvironments of the high-risk and low-risk groups.

**Figure S5.** Comparison of HLM Score and CMS.

**Figure S6.** ROC curves, risk heatmaps, and calibration curves in the validation sets.

**Figure S7.** scRNA-seq analysis to assess immune cell infiltration.


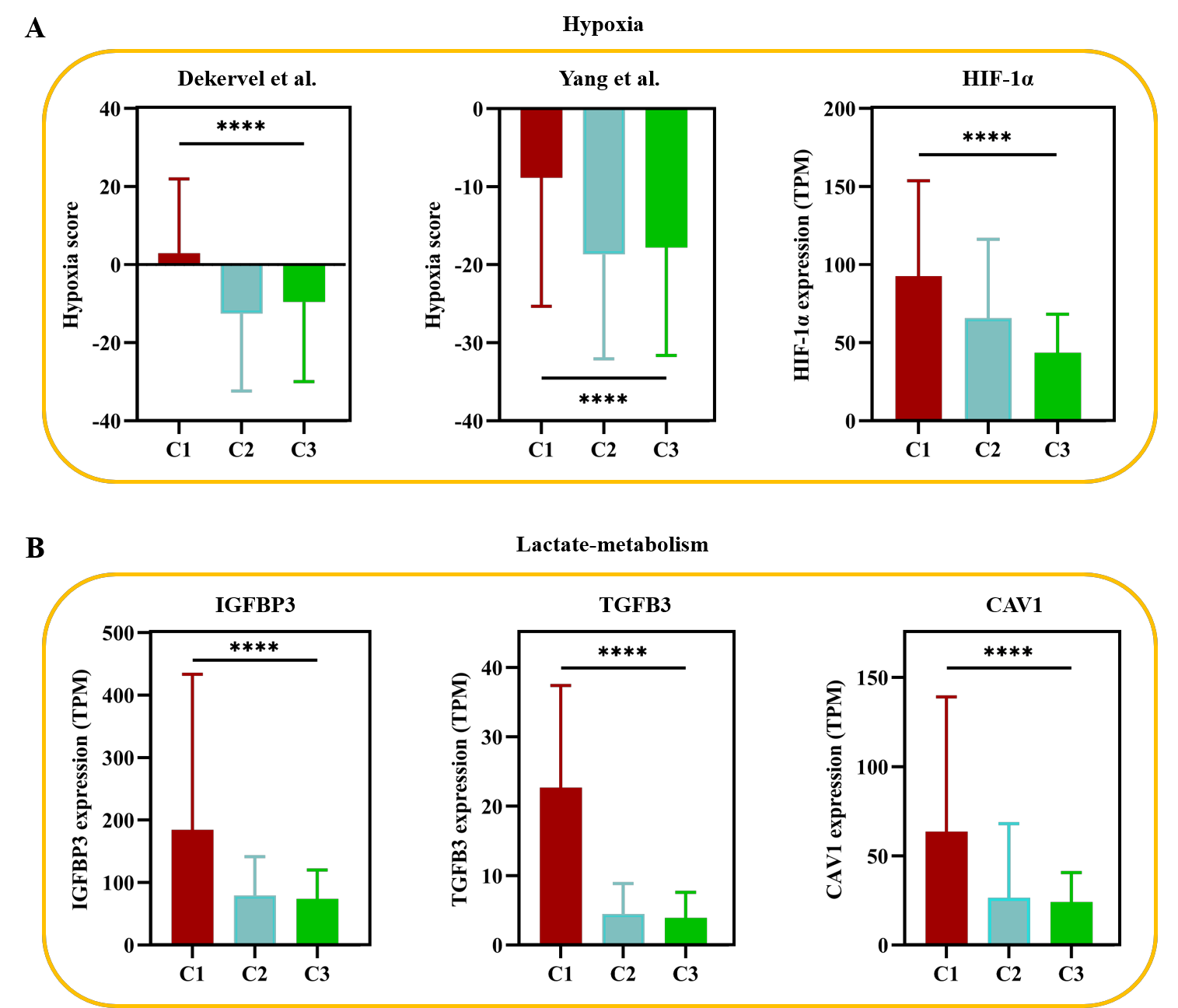


**Figure S1.** Hypoxia- and lactate metabolism-related microenvironments of the three subtypes. **(A)** Hypoxia microenvironment. **(B)** Lactate metabolism-related microenvironment.


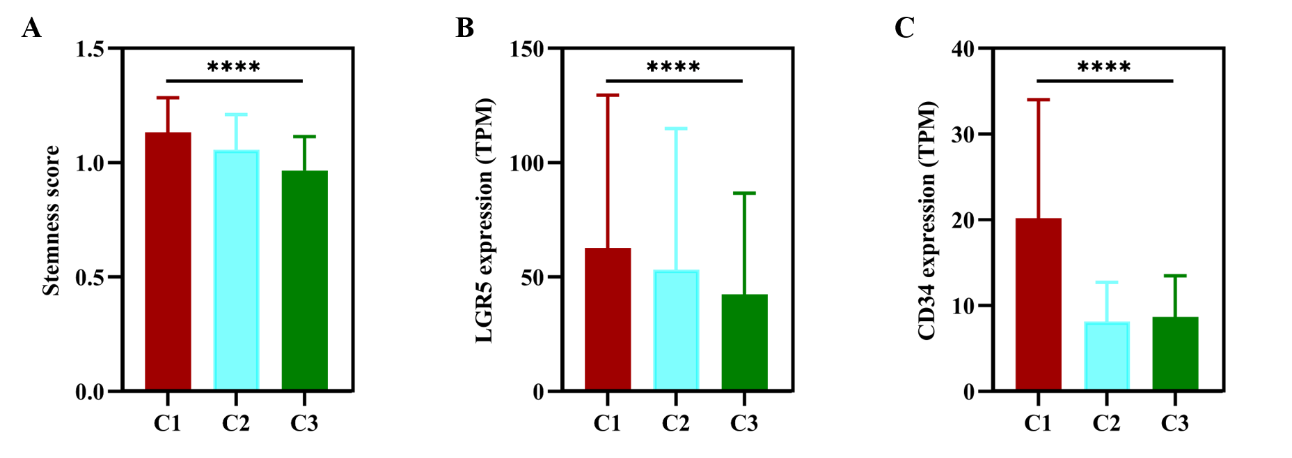


**Figure S2.** Stemness enrichment scores and expression of stemness-associated genes in different subtypes. **(A)** Stemness enrichment scores. **(B)** Expression of LGR5. **(C)** Expression of CD34.


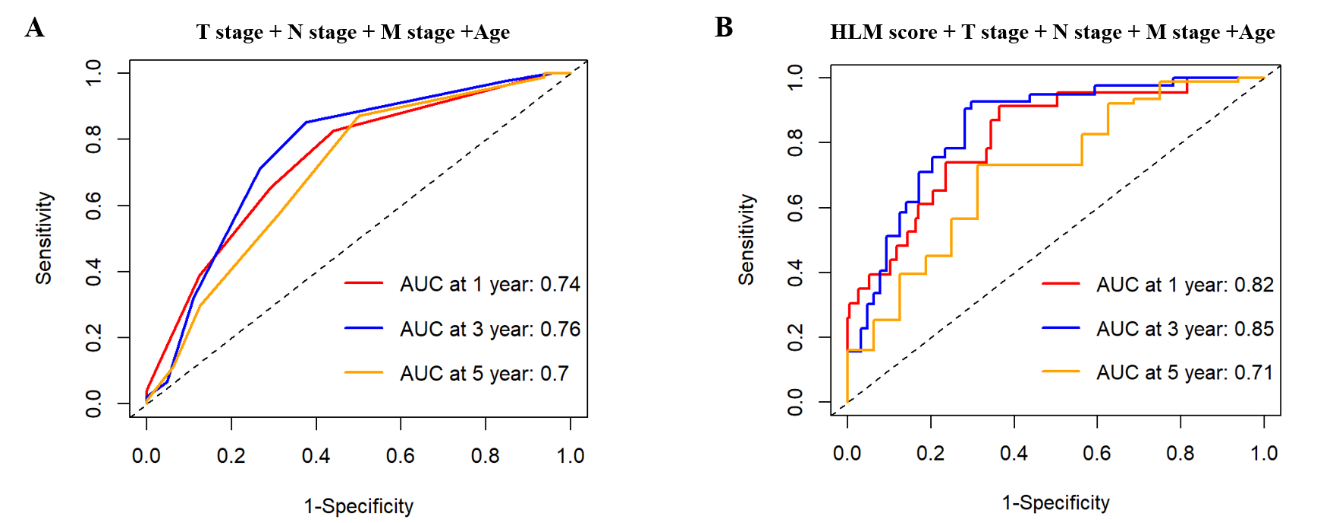


**Figure S3.** Time-dependent ROC curves for predicting 1-, 3-, and 5-year OS. **(A)** Clinical factors alone. **(B)** The HLM score combined with clinical factors.


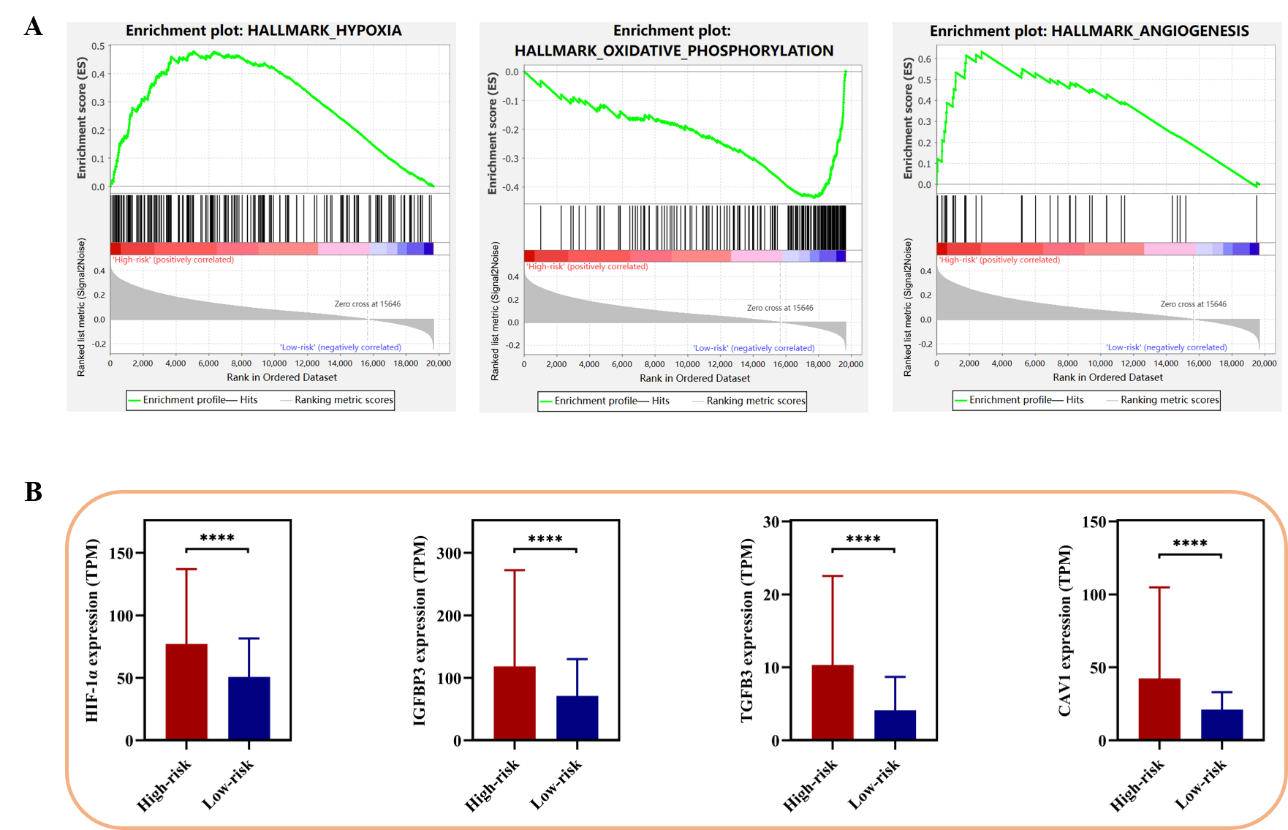


**Figure S4.** Hypoxia- and lactate metabolism-related microenvironments of the high-risk and low-risk groups. **(A)** GSEA of the high-risk and low-risk groups. **(B)** Hypoxia- and lactate metabolism-related microenvironment of the high-risk and low-risk groups.


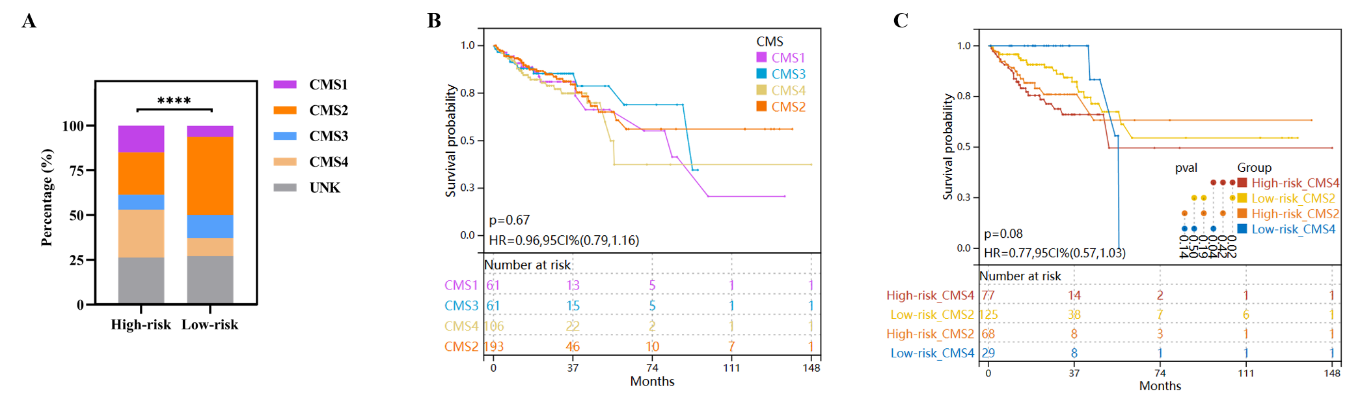


**Figure S5.** Comparison of HLM Score and CMS. **(A)** Distribution of the CMS in the high-risk and low-risk group. **(B)** CMS for CRC prognostic stratification. **(C)** HLM score combined with CMS for CRC prognostic stratification.


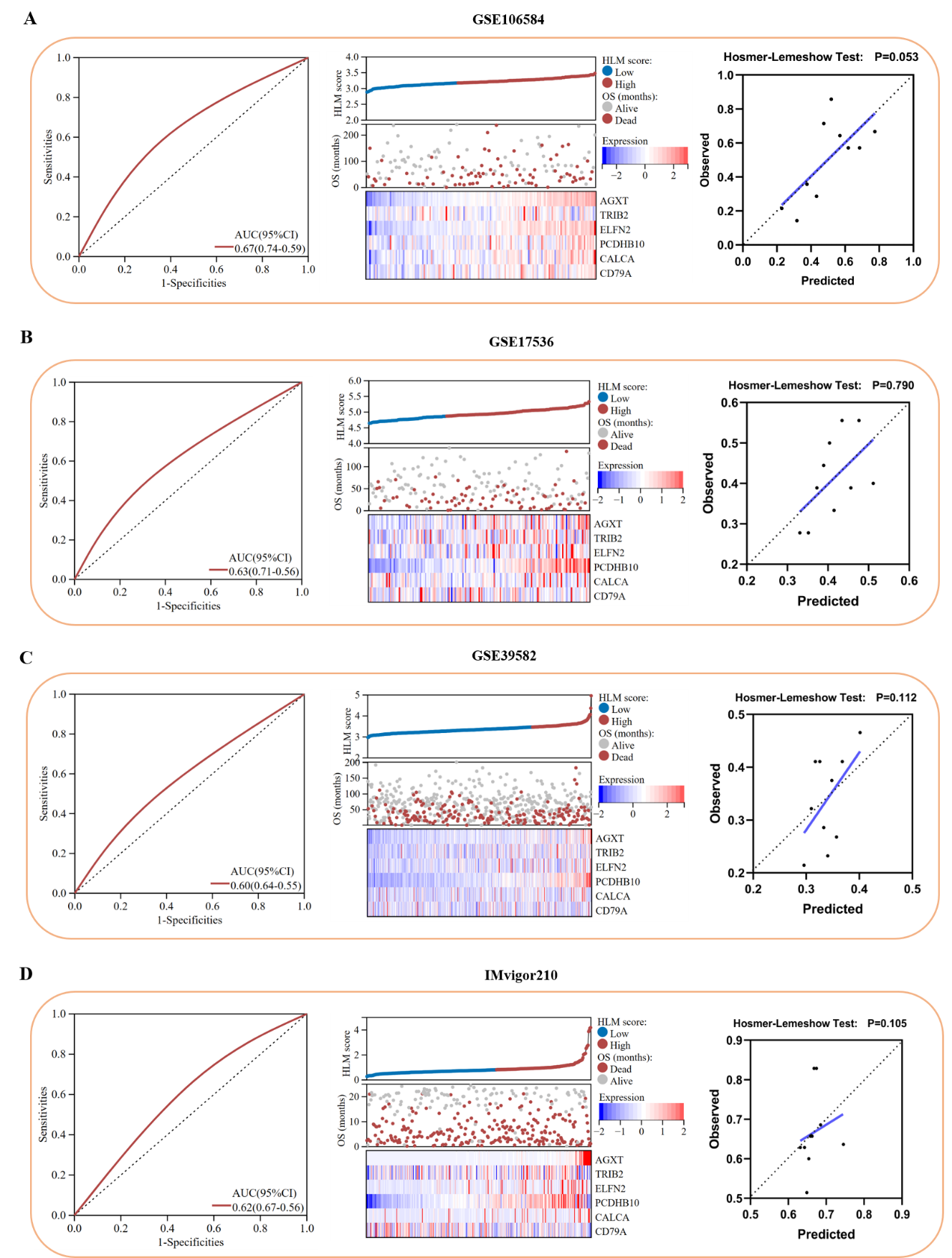


**Figure S6.** ROC curves, risk heatmaps, and calibration curves in the validation sets. **(A)** GSE106584, **(B)** GSE17536, **(C)** GSE39582 and **(D)** IMvigor210.


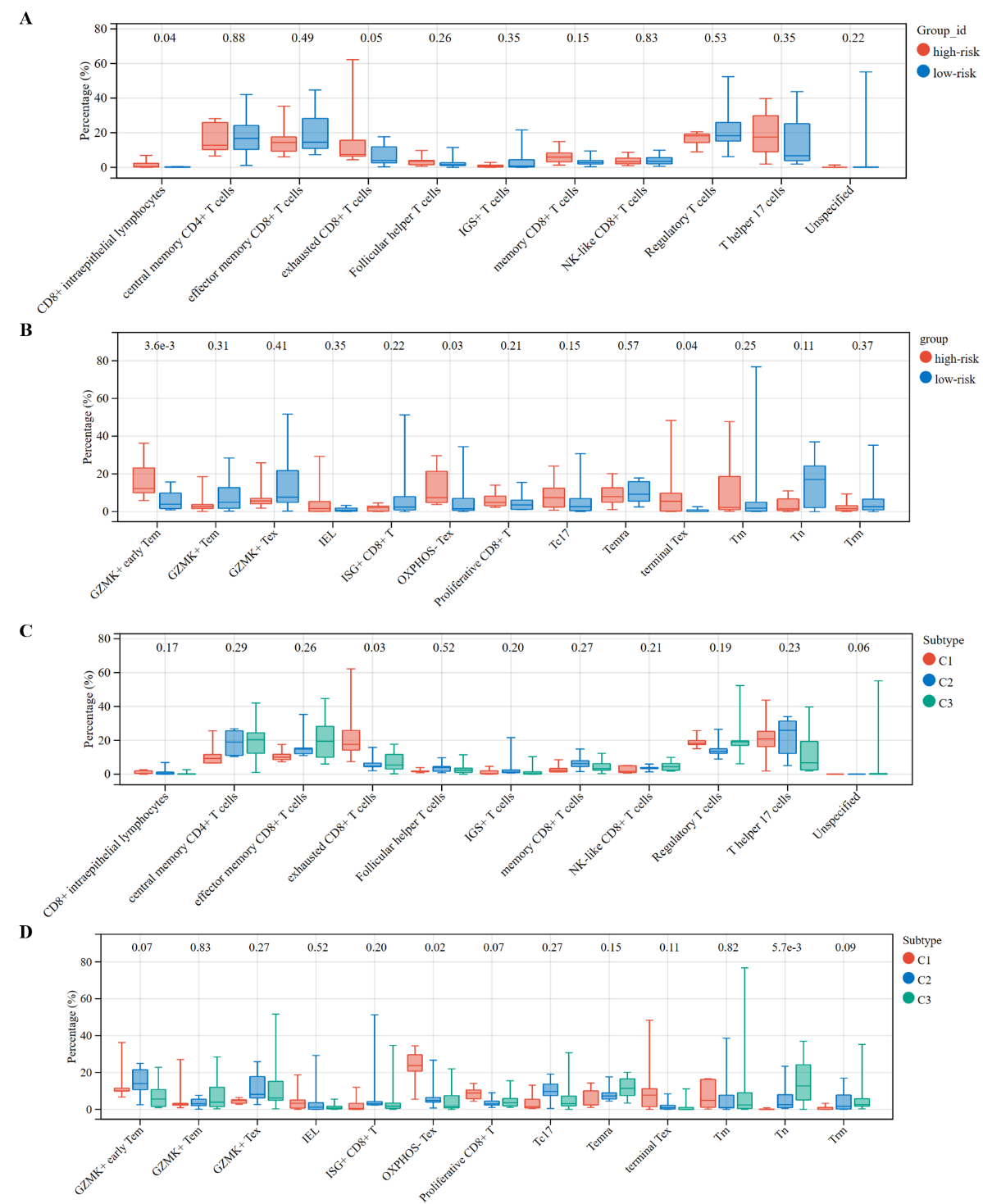


**Figure S7.** scRNA-seq analysis to assess immune cell infiltration. **(A)** Percentage of T cell subpopulation infiltration in high-risk vs. low-risk groups. **(B)** Percentage of CD8+ T cell subpopulation infiltration in high-risk vs. low-risk groups. **(C)** Percentage of T cell subpopulation infiltration in different molecular subtypes. **(D)** Percentage of CD8+ T cell subpopulation infiltration in different molecular subtypes. Tem, effective memory T cells; IEL, intraepithelial lymphocyte; Tc17, IL-17-producing CD8+ T cells; Tm, memory T cells; Tn, naïve T cells; Trm, tissue-resident memory T cells.
